# Rapid and large-scale glycopeptide enrichment strategy based on chemical ligation

**DOI:** 10.1093/nsr/nwae341

**Published:** 2024-09-27

**Authors:** Yingying Xiong, Zhuoer Lu, Yuyin Shao, Peiyi Meng, Guoli Wang, Xinwen Zhou, Jun Yao, Huimin Bao, Haojie Lu

**Affiliations:** Department of Chemistry and Liver Cancer Institute, Zhongshan Hospital, Fudan University, Shanghai 200032, China; Department of Chemistry and Liver Cancer Institute, Zhongshan Hospital, Fudan University, Shanghai 200032, China; Institutes of Biomedical Sciences and NHC Key Laboratory of Glycoconjugates Research, Fudan University, Shanghai 200032, China; Department of Chemistry and Liver Cancer Institute, Zhongshan Hospital, Fudan University, Shanghai 200032, China; Institutes of Biomedical Sciences and NHC Key Laboratory of Glycoconjugates Research, Fudan University, Shanghai 200032, China; Institutes of Biomedical Sciences and NHC Key Laboratory of Glycoconjugates Research, Fudan University, Shanghai 200032, China; Institutes of Biomedical Sciences and NHC Key Laboratory of Glycoconjugates Research, Fudan University, Shanghai 200032, China; Department of Chemistry and Liver Cancer Institute, Zhongshan Hospital, Fudan University, Shanghai 200032, China; Department of Chemistry and Liver Cancer Institute, Zhongshan Hospital, Fudan University, Shanghai 200032, China; Institutes of Biomedical Sciences and NHC Key Laboratory of Glycoconjugates Research, Fudan University, Shanghai 200032, China

**Keywords:** glycoproteomics, large-scale enrichment, O-GlcNAcylation, N-glycosylation, oxidative stress

## Abstract

Protein glycosylation, the most universal post-translational modification, is thought to play a crucial role in regulating multiple essential cellular processes. However, the low abundance of glycoproteins and the heterogeneity of glycans complicate their comprehensive analysis. Here, we develop a rapid and large-scale glycopeptide enrichment strategy via bioorthogonal ligation and trypsin cleavage. The enrichment process is performed in one tube to minimize sample loss and time costs. This method combines convenience and practicality, identifying over 900 O-GlcNAc sites from a 500 μg sample. Surprisingly, it allows simultaneous identification of N-glycosites, O-GlcNAc sites, O-GalNAc sites and N-glycans via a two-step enzymatic release strategy. Combined with quantitative analysis, it reveals the distinct O-GlcNAcylation patterns in different compartments during oxidative stress. In summary, our study offers a convenient and robust tool for glycoproteome and glycome profiling, facilitating in-depth analysis to elucidate the biological functions of glycosylation.

## Teaser

This study introduces a convenient method for rapidly enriching and analyzing glycopeptides, enabling the simultaneous identification of multiple glycosylation types and revealing new insights into O-GlcNAcylation during oxidative stress.

## INTRODUCTION

Protein glycosylation is the most universal post-translational modification, and regulates fundamental cellular processes ranging from protein folding and trafficking to signal transduction and metabolism [1–3]. There are various types of glycosylation, including N-glycosylation, O-GlcNAcylation and mucin-type O-glycosylation. In recent years, liquid chromatography-tandem mass spectrometry (LC-MS/MS) has become a powerful tool for protein modification profiling [4,5]. However, due to the low abundance of glycoproteins and the heterogeneity of glycans, it is necessary to develop enrichment approaches before MS analysis. For N-glycopeptide enrichment, hydrophilic interaction liquid chromatography (HILIC) is a very convenient and commercially available tool [6,7]. However, large-scale O-glycoproteome characterization has always been challenging. Xiao *et al*. developed an enrichment strategy using dendrimer-conjugated benzoboroxole, but the scale of the O-glycopeptide identified is still limited [8]. In addition, most enrichment methods are limited to individual glycosylation types, and methods for N-glycopeptides and O-glycopeptides involve different experimental conditions. Even between the technical replicates, guaranteeing identical results remains challenging. So, in comprehensive analysis, mapping them individually may introduce operational and technical variations that lead to inconsistent data, especially for certain dynamic and complex biological models or systems. Therefore, it is necessary to report a universal and large-scale enrichment strategy for multiple types of glycosylation.

Currently, due to the high efficiency of click chemistry and high-affinity interaction between biotin and streptavidin, methods relying on the introduction of bioorthogonal groups and biotin-avidin strategies to enrich glycoproteins have been popular, especially the Click-iG platform [9–13]. This integrates biotin-avidin strategies with an optimized MS method and a tailored version of pGlyco3 software to analyze all types of intact glycopeptides simultaneously within a single experiment, which provides valuable data sets for a comprehensive understanding of protein glycosylation. However, these strategies require complex sample preparation (biotin labeling, biotin-avidin capture, release step and methanol-chloroform precipitation to remove excess biotin) and considerable amounts of sample. Moreover, the biotin-avidin platform is limited by the interference of endogenous biotinylated proteins. Recently, Chen *et al*. reported a reversible chemoenzymatic enrichment approach by Endo-M mutant labeling and HILIC enrichment, but it is difficult to obtain Endo-M [14]. Therefore, we intend to develop a novel tool for comprehensive profiling of multiple types of glycosylation with easy operation and accessible materials or reagents.

**Figure 1.**
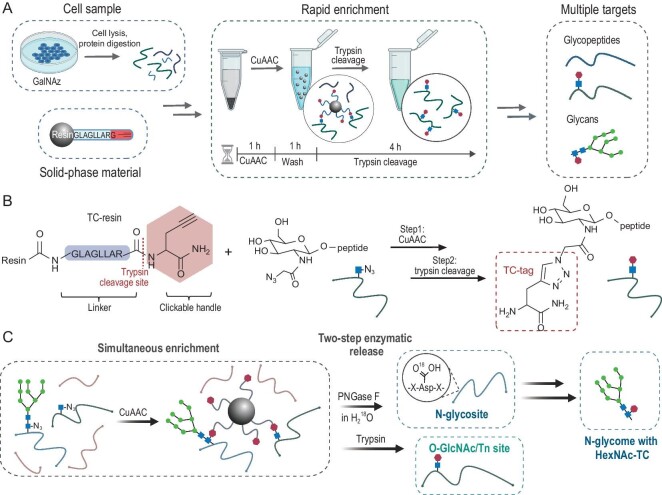
The rapid and large-scale HexNAc-containing glycopeptide and glycan enrichment strategy based on TC-resins. (A) Workflow of the rapid and large-scale enrichment strategy based on TC-resins. (B) Schematic illustration of TC-resins for HexNAz-containing peptide enrichment. The rectangle, GlcNAc. The hexagon, the clickable handle. The structure in the dotted rectangle is the TC-tag. (C) Schematic illustration of simultaneous identification of N-glycosites, O-GlcNAc sites, O-GalNAc sites and N-glycome via a two-step enzymatic release strategy.

Here, inspired by the design of cleavable biotin and the previous resin-assisted approach our group took [15,16], we describe a rapid and large-scale N-acetylhexosamine (HexNAc)-containing glycopeptide enrichment strategy based on the solid-phase material (trypsin cleavage (TC)-resin), namely HG-TCs (Fig. 1A). The solid-phase material is functionalized with an alkyne group for capture and with a trypsin-cleavable linker for release (Fig. 1B). After being labeled with an azide group by metabolic labeling, glycopeptides are attached to TC-resins via bioorthogonal ligation and released by efficient trypsin cleavage. The enrichment process can be completed in one tube within 6 h to avoid sample loss and time costs. We first proposed this idea to enrich the O-GlcNAc peptides and identified over 900 O-GlcNAc glycosites from a 500 μg sample in a single run. Surprisingly, this method allows simultaneous identification of N-glycosites, O-GlcNAc sites, O-GalNAc (Tn-antigen) sites and N-glycans via the two-step enzymatic release strategy (Fig. 1C). In total, we identified 2028 O-GlcNAc sites on 462 O-GlcNAc proteins, 855 N-glycosites on 445 N-glycoproteins, and 71 Tn-antigen glycosites on 37 Tn-antigen glycoproteins. We also obtained 98 N-glycans with TC-tags, providing the N-glycome for more comprehensive information. By integrating this method with the tandem mass tag (TMT) labeling strategy, we revealed the distinct spatial O-GlcNAcylation patterns between the nucleus and cytoplasm during oxidative stress for the first time. In conclusion, our study offers a convenient and robust strategy for glycopeptide enrichment, facilitating comprehensive proteomic analysis and advancing our understanding of protein glycosylation.

## RESULTS AND DISCUSSION

### Large-scale O-GlcNAc glycoproteome characterization by HG-TCs

Due to the low abundance of O-glycopeptides compared to N-glycopeptides, large-scale O-GlcNAc glycoproteome characterization has been a difficult task. As shown in Fig. 1A, we designed the rapid and large-scale HexNAc-containing glycopeptide enrichment strategy (HG-TCs) based on TC-resin and attempted to use this approach for O-GlcNAc peptide enrichment. TC-resin is a solid-phase material functionalized with an alkyne group and trypsin-cleavable linker. In this strategy, we first obtain all peptides (both azide-labeled and unlabeled) from cell samples through metabolic labeling and protein digestion. For the enrichment step, azide-labeled glycopeptides are attached to TC-resins via Cu^I^-catalyzed azide-alkyne cycloaddition (CuAAC), while other peptides are removed by stringent washes. Finally, in the release step, the solid TC-resin is resuspended in a trypsin enzymatic system. Since the linker peptide between glycopeptide and resin contains arginine and trypsin specifically hydrolyzes the carboxyl side of arginine, the glycopeptide can be released by trypsin. When trypsin hydrolyzes the carboxyl side of arginine, the ‘clickable handle’ part attaches to the glycopeptide along with the five-membered ring formed in the CuAAC step (Fig. S1 in Supplementary Data), which introduces an amino group to facilitate the protonation of glycopeptides during MS analysis [15]. The integral part of the five-membered ring, formed by the alkyne handle and the azide group, is called the TC-tag. Compared to the workflow of the biotin-based approach, our procedure can omit the introduction of biotin and methanol-chloroform precipitation to provide a simpler workflow (Fig. S2). Crucially, our enrichment process can be accomplished in one tube within 6 h, offering the same convenience as HILIC and significantly minimizing sample loss, labor and time costs.

**Figure 2.**
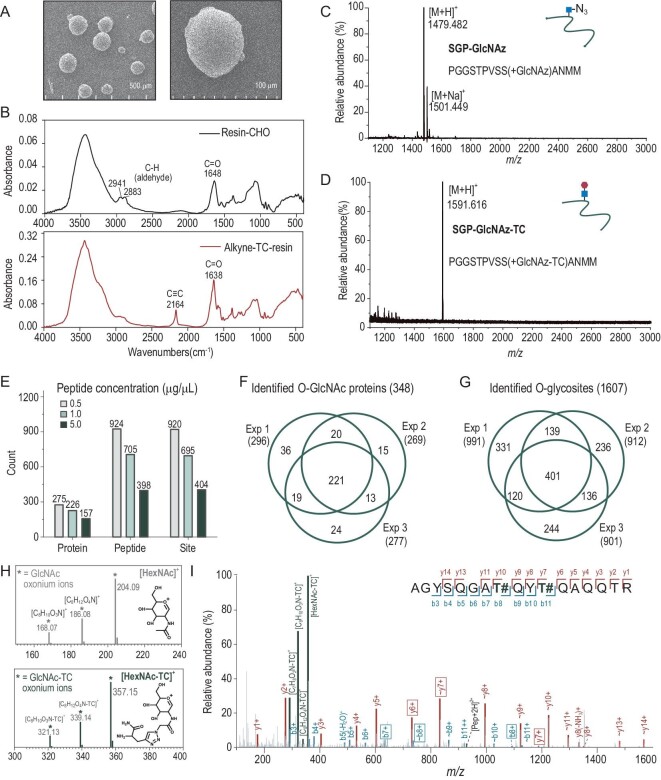
The successful application of TC-resin in large-scale O-GlcNAc proteomic profiling from cell samples. (A and B) Representative SEM images (A) of TC-resins and the FT-IR curves (B) of aldehyde resins (top) and TC-resins (bottom). (C and D) MALDI-TOF-MS spectra of the synthetic O-GlcNAz peptide (C) and the released peptide (D) after enrichment. (E) Numbers of identified glycoproteins, glycopeptides and glycosites with different peptide concentrations (0.5, 1.0 and 5.0 μg/μL). (F and G) Venn diagrams of O-GlcNAc proteins (F) and glycosites (G) identified by triplicate experiments from HeLa nuclear samples. (H) Scheme of the GlcNAc and TC-tagged GlcNAc oxonium ions. (I) HCD MS/MS spectrum of a glycopeptide containing two glycosites on ZFR (Thr199 and Thr202 as the O-GlcNAc sites).

The successful synthesis of material is a prerequisite for enrichment. TC-resins can be easily prepared via a reductive amination reaction between aldehyde resin and alkyne peptide (GLAGLLARG-alkyne) in the presence of NaBH_3_CN (Fig. S3). Agarose-based resins were depicted through high-resolution scanning electron microscope (SEM) images, showing an average diameter of ∼500 μm, and were uniform both in shape and in size (Fig. 2A). The successful synthesis of TC-resin was proven by various methods. First, the surface modification of aldehyde resin and subsequent decoration with alkyne peptide were monitored using Fourier transform infrared (FT-IR) spectra (Fig. 2B). Characteristic peaks at 2883 cm^−1^ and 2941 cm^−1^, indicating Fermi resonance from the C–H bond of the aldehyde group, were observed. After coating with a layer of alkyne peptides, an additional peak at 2164 cm^−1^ appeared, attributed to the triple bond from the alkyne group. Simultaneously, supernatants after the reaction, both with and without the presence of NaBH_3_CN, were detected through matrix-assisted laser desorption ionization time-of-flight mass spectrometry (MALDI-TOF-MS) (Fig. S4). A peak of alkyne peptide was observed in the supernatant without NaBH_3_CN incubation, and disappeared after the addition of NaBH_3_CN. These findings indicated the successful conjugation of alkyne peptide to resin, and that the conjugation was formed by a reductive amination reaction rather than by non-specific adsorption.

Next, we used a model of synthetic O-GlcNAz peptide (SGP-GlcNAz, *m/z* = 1479.48 Da, as shown in Fig. 2C) to evaluate the performance of TC-resin. After the CuAAC reaction, the peak of SGP-GlcNAz nearly disappeared in the supernatant, indicating the high efficiency of the capture step (Fig. S5A). After trypsin cleavage, the released glycopeptide (SGP-GlcNAz-TC, *m/z* = 1591.61 Da) was labeled by an amine-containing tag with an additional mass gain of 112.13 Da (Fig. 2D). These results showed that O-GlcNAz peptides can be enriched successfully by TC-resin. Then, we investigated whether TC-resin can be used for O-GlcNAc proteomic profiling from cell samples. First, taking into account the interference from artificial S-glycosylation, we chose N-azidoacetyl-galactosamine (GalNAz) for metabolic labeling [17,18]. HeLa cells were cultured with 500 μM GalNAz for 48 h. The successful labeling of proteins in HeLa cells was confirmed by in-gel fluorescence imaging (Fig. S5B). Subsequently, to exclude the interference of O-GalNAc proteins, and considering that O-GlcNAc proteins are mainly located in the nucleus, we isolated nucleus proteins for further enrichment [14]. Western blotting experiments verified the effective separation of the nucleus from HeLa cells (Fig. S5C).

According to previous reports, azide groups are easily reduced to amino groups, especially in the presence of dithiothreitol (DTT) generally used in reduction and alkylation steps [19,20]. We investigated the stability of azide-peptides under denaturing conditions, including DTT and thermal denaturation (Fig. S6). Results indicated that over 95% of azide-peptides were reduced under general reductant conditions (10 mM DTT for 30 min), whereas they exhibited greater stability under heat-denaturing conditions (75°C for 30 min). So, we adopted 75°C for 30 min as the denaturing condition before protein digestion. In our preceding research, we have demonstrated that the cleavable linkers are easily cleaved during on-bead digestion [15]. Due to the potential persistence of tryptic activity after lysate digestion, it is imperative to ensure that the linkers are not cleaved during the enrichment. The linker peptides were incubated with trypsin-treated lysates for 4 h, and the result showed that the linkers were cleaved, with only ∼0.5% of linkers remaining intact (Fig. S7A and B). Subsequently, when subjecting the same lysates to a 75°C treatment for 20 min before incubation with linker peptides, nearly 98% of linkers remained intact (Fig. S7C). These results demonstrated that residual tryptic activity must be removed before enrichment, and heat treatment is a straightforward and efficacious solution.

During the enrichment step, glycopeptides were directly captured by TC-resin via click chemistry. The challenge of this step arises from the fact that the click reaction occurs between the alkyne on solid-phase material and the azide-peptide in solution, differing from the normal click reaction in a liquid environment. To enhance the enrichment efficiency, we systematically optimized both peptide concentration and resin quantity in the click reaction using 500 μg of starting samples. First, 500 μg of peptides was mixed with different amounts of TC-resin (10, 20 and 30 μL) at a peptide concentration of 0.5 μg/μL (Fig. S8; Table S1). Then, peptides at different concentrations (0.5, 1.0 and 5.0 μg/μL) were mixed with 20 μL of resin (Fig. 2E; Table S1). The results indicated that the optimal conditions involved 500 μg of starting samples, 20 μL of resin, and a peptide concentration of 0.5 μg/μL. Then we used the optimal conditions for O-GlcNAc proteomic profiling. In triplicate experiments, ∼900 potential glycosites on 300 glycoproteins (991, 912 and 901 sites from 296, 269 and 278 glycoproteins, respectively) were identified from 500 μg of nuclear samples in a single LC-MS/MS run (Fig. 2F and G; Table S2). About 80% of the glycoproteins and 60% of the glycosites were consistently identified in at least two of the triplicates, suggesting the good repeatability of this approach. Integrating results from the above triplicates, a total of 348 O-GlcNAc proteins with 1607 potential glycosites were identified in this study (Table S3). The above results indicated that HG-TCs combine convenience and practicality, allowing large-scale O-GlcNAc glycoproteome characterization in cell samples with a simple workflow and low-input samples.

### The trypsin cleavage tag facilitates O-GlcNAc peptide identification and O-GlcNAc site confirmation

To further corroborate the glycopeptide and validate O-GlcNAc sites, we scrutinized the fragments in the MS/MS spectra of identified glycopeptides. In previous studies, higher-energy collisional dissociation (HCD) is effective in generating GlcNAc oxonium ions at *m/z* 204.08 from O-GlcNAc peptide precursor ions, distinguishing them from non-glycosylated peptides [21]. In our results, several specific oxonium ions were observed, particularly the oxonium ion at *m/z* 357.15, which represents the intact TC-tagged GlcNAc moiety (Fig. 2H). It was observed in the MS/MS spectra of all identified glycopeptides and can be used as a diagnostic peak for O-GlcNAc peptide identification. Moreover, due to the low normalized collision energy (28%) we used, the fragment ions containing residual glycan can be observed. When the break position of the glycan-containing fragment ion is proximal to the candidate site, this fragment contributes to the unambiguous localization of the O-GlcNAc site (Fig. S9A). Approximately 89% of MS/MS spectra contained fragment ions with residual glycan. Of these spectra, ∼70% contained ions that enabled unambiguous site localization (Fig. S9B). For instance, two glycopeptides with the same peptide sequence were detected from the nuclear pore complex protein Nup214 (Fig. S9C). Although this sequence has eight S or T residues, the glycan-containing fragment ions enabled precise localization of O-GlcNAc sites on Thr1055 (y_4_^+^ and y_5_^+^) and Ser1056 (y_3_^+^ and y_4_^+^), respectively. Additional evidence supporting glycosite confirmation was obtained through the fragment ions with glycan loss (∼y_4_^+^ and ∼y_5_^+^).

Importantly, this approach enabled the recognition and labeling of O-GlcNAc moieties at the peptide level to reduce steric hindrance during CuAAC, which enhances the capacity to capture glycosites and has an advantage in capturing proteins or peptides containing multiple O-GlcNAc sites (Fig. S10). For example, a glycopeptide containing two glycosites (AGYSQGAT#QYT#QAQQTR, where # denotes the glycosite) from zinc finger RNA-binding protein (ZFR) was observed (Fig. 2I). Intense oxonium ions of TC-tagged GlcNAc were observed, and the y_7_^+^ and b_8_^+^ ions from the MS/MS spectrum successfully localized the glycosites of Thr199 and Thr202 in one peptide. In brief, this method can provide comprehensive information for O-GlcNAc peptide identification and O-GlcNAc site confirmation based on the TC-tag.

### Simultaneous identification of N-glycosites, O-GlcNAc sites, O-GalNAc sites and N-glycans by two-step enzymatic release

Due to the shared utilization of the same nucleotide sugars in different glycan biosyntheses, the metabolic glycan labeling strategy proved versatile for various glycoprotein types. Benefiting from the salvage pathway, GalNAz can be converted to UDP-GalNAz, interchangeably transformed with UDP-GlcNAz by UDP-galactose-4-epimerase (GALE). As a result, various types of glycoproteins are metabolically labeled with azides, including N-glycoproteins and O-glycoproteins [13,22,23]. Leveraging this principle, we devised a simultaneous enrichment method employing a two-step enzymatic release strategy to obtain N-glycosites, O-GlcNAc sites and O-GalNAc (the Tn antigen) sites simultaneously (Fig. 1C). The initial steps and experimental conditions are the same as the O-GlcNAc procedure, but in the release step, we first performed on-bead digestion with PNGase F under heavy-oxygen water (H_2_^18^O) to release peptides containing N-glycosites. Subsequently, O-glycopeptides were released by trypsin cleavage. Interestingly, the N-glycans were still attached to the beads after PNGase F treatment and were retained in O-glycopeptide fractions after trypsin cleavage. So, the flow-through and wash buffer after peptide desalting were collected for cotton enrichment to obtain N-glycans with HexNAc-TC [24], which enabled the identification of the metabolically labeled N-glycome (Fig. S12A). In addition, we can also obtain the information of the Tn antigen. The O-GlcNAc and the Tn antigen have identical chemical compositions but differ only in spatial conformation, which makes it challenging to distinguish them. To ensure the reliability of the data, we referred to the previous study to assign the O-GlcNAc and Tn antigen manually based on the subcellular localization in UniProt [13].

**Figure 3.**
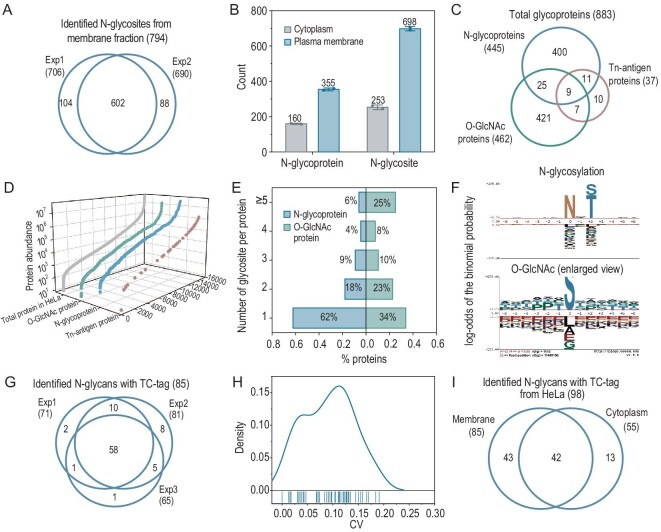
Simultaneous identification of N-glycosites, O-GlcNAc sites, O-GalNAc sites and N-glycome via the two-step enzymatic release strategy. (A) Venn diagram of N-glycosites identified by two replicates from membrane fractionated samples. (B) Numbers of N-glycosites and N-glycoproteins identified from membrane and cytoplasm samples. Error bars represent mean *±* s.d. from two independent experiments. Average values are shown above the bars. (C) Overlap of identified N-glycoproteins, O-GlcNAc proteins and Tn-antigen proteins. (D) Copy number distribution of the identified N-glycoproteins, O-GlcNAc and Tn antigen proteins compared with the total HeLa protein database. (E) Distribution of glycosite number per glycoprotein. (F) Sequence analysis around the N-glycosites and O-GlcNAc sites. (G) Venn diagram of N-glycans with TC-tag identified by three replicates from membrane fraction. (H) The variation coefficient of N-glycans with TC-tag identified by three replicates. (I) Venn diagram of N-glycans with TC-tag identified from membrane and cytoplasm.

For this method, we utilized membrane and cytoplasm samples from the previous fractionation. From membrane samples, a total of 794 N-glycosites from 395 N-glycoproteins were identified (Fig. 3A and S11A). Approximately 90% of glycoproteins and 87% of glycosites were consistently identified in two replicates, showing the great repeatability of this approach. We also obtained 71 Tn-antigen glycosites from 37 potential Tn-antigen glycoproteins and 805 potential O-GlcNAc sites from 255 O-GlcNAc proteins (Fig. S11B and C). For N-glycosylation and Tn antigen, the numbers of glycosites and glycoproteins were higher in the membrane samples than in the cytoplasm, which is consistent with their localization (Fig. 3B and Fig. S11C). Combining all results, we identified 445 N-glycoproteins, 462 O-GlcNAc proteins and 37 Tn-antigen glycoproteins with their glycosite information (Fig. 3C; Tables S4–S9). Compared with all proteins in the HeLa protein database [25], the abundance patterns covered 6∼7 orders of magnitude (Fig. 3D), indicating that this strategy allows for the high coverage of glycoproteins identification. In addition, ∼25% of O-GlcNAc proteins were identified with at least five glycosites. However, most N-glycoproteins possessed a single site, with only 6% containing at least five sites (Fig. 3E). Sequence analysis around the glycosites revealed the typical motif for N-glycosylation and the lack of a strict consensus sequence in O-GlcNAcylation (Fig. 3F and Fig. S11D), consistent with the view that N-acetylglucosamine transferase (OGT) primarily binds to substrates through contact with the peptide backbone rather than specific side chain [26,27]. Interestingly, we noted a preference for serine/threonine-rich stretches from the sequence in O-GlcNAcylation, potentially explaining the high proportion of O-GlcNAc site-enriched glycoproteins identified in our study.

For the N-glycome profiling, 85 N-glycans with TC-tag were identified by three replicates from membrane fraction (Fig. 3G; Table S10). The density distribution of the coefficient variation showed good reproducibility and stability across replicates (Fig. 3H). For the N-glycan type, the complex type was the most abundant (Fig. S12B). In addition, since the N-glycans contain multiple HexNAc units, the same glycan composition may carry multiple TC-tags, which may complicate the analysis. We found that most of the N-glycans are labeled with a single TC-tag (Fig. S12C). To reveal the preferences of HexNAz incorporation at the glycan type level, we classified N-glycans according to the number of tags and found a similar proportion of sialic acid-containing N-glycans. In total, we identified 98 N-glycans with TC-tags from membrane and cytoplasm fractions (Fig. 3I). We also analyzed the distribution of N-glycans with specific types in different compartments, and found similar distribution patterns of glycan types for the N-glycans labeled with a single TC-tag (Fig. S12D).

### Comprehensive analysis of the O-GlcNAc proteome in the HeLa nucleus

**Figure 4.**
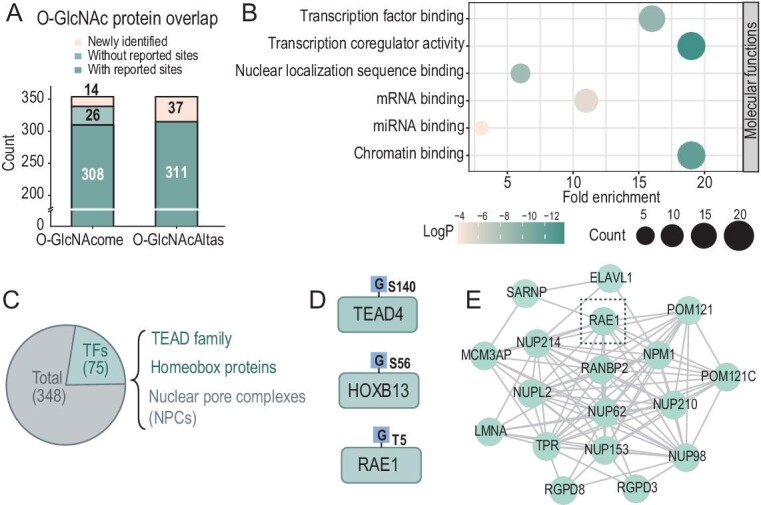
In-depth analysis of O-GlcNAc proteome in a HeLa cell. (A) Overlap of identified glycoproteins with two published O-GlcNAc databases. (B) GO analysis of site-enriched O-GlcNAc proteins (at least five glycosites) based on molecular functions. (C) Distribution of O-GlcNAc proteins annotated as transcription factors (TFs). (D) O-GlcNAc sites of TEAD4, HOXB13 and RAE1 identified in this study. (E) Interaction network of identified O-GlcNAc proteins involved in nucleocytoplasmic transport. Each node stands for a protein, and each edge represents a known protein–protein interaction.

Subsequently, we sought to provide a novel contribution by comparing our data from the nucleus with two recent O-GlcNAc databases: the O-GlcNAcome and the O-GlcNAcAtlas (Fig. 4A; Table S3) [28,29]. Compared with the O-GlcNAcome, 96% (334/348) of the identified glycoproteins and 63% (1009/1607) of the glycosites in our data were covered, providing 14 novel O-GlcNAc proteins and 598 novel glycosites. Similarly, a comparison with the O-GlcNAcAtlas revealed that 89% (311/348) of the glycoproteins and 56% (909/1607) of the glycosites were cataloged in the database, respectively. Gene ontology (GO) analysis showed that the identified O-GlcNAc proteins were mainly localized in the nucleus, aligning with the sample preparation (Fig. S13). As mentioned above, many O-GlcNAc proteins were site-enriched (at least five glycosites), so we analyzed these glycoproteins and found that they have functions related to chromatin binding and transcription co-regulatory activity (Fig. 4B).

Given the intimate association between O-GlcNAcylation and transcription, 20% (75/348) of the glycoproteins were annotated as transcription factors (TFs) (Fig. 4C; Table S11) [30], including TEAD4 and HOXB13, previously lacking reported O-GlcNAc sites (Fig. 4D). The TEAD family TFs that are essential for mediating YAP-dependent gene expression [31] were identified in our results, including TEAD1, TEAD3 and TEAD4 (Figs S14 and S15). Among them, TEAD4 had no mapped O-GlcNAc sites yet. We identified Ser140 as the O-GlcNAc site on TEAD4 (Fig. S15A). Likewise, HOXB13 lacked O-GlcNAcylation annotation in the UniProt, but we identified it with high scores given by Byonic and confirmed Ser56 as the O-GlcNAc site (Fig. S15B). We also observed diverse protein–protein interactions, including the regulation of RNA splicing, stress granule assembly and nucleocytoplasmic transport (Fig. S16; Table S7) [32]. Nucleocytoplasmic transport can affect the subcellular localization of many proteins, which is essential for the proper functioning of eukaryotic cells. The nuclear-cytoplasm trafficking is mainly controlled by nuclear pore complexes (NPCs), composed of multiple nucleoporins (Nups) [33]. Several Nups annotated as highly O-GlcNAc-modified were observed in our data, including NUP62, NUP214, NUP153, POM121, NUP210 and NUP98 (Fig. 4E) [34]. Notably, RAE1, a component of the NPCs lacking definite O-GlcNAc sites in the database [35], was found in our data with the O-GlcNAc site Thr5 (Fig. S17). This comprehensive information on O-GlcNAcylation in Nups may contribute to elucidating the mechanisms underlying nucleocytoplasmic transport.

### Quantitative proteomic analysis revealed the distinct spatial O-GlcNAcylation patterns between the nucleus and cytoplasm during oxidative stress

Numerous studies have demonstrated that protein O-GlcNAcylation is a conserved intracellular signaling mechanism activated by diverse stressors such as heat shock, osmotic stress and oxidative stress [36,37]. Some stressors increased protein O-GlcNAcylation in a dose-dependent manner, some stressors decreased, and some displayed complex dynamics in O-GlcNAcylation cycling [38,39]. Zachara's group revealed that O-GlcNAcylation levels in mouse embryonic fibroblasts (MEFs) under oxidative stress changed from decrease to increase over time [40]. These studies have focused on changes in O-GlcNAcylation at global levels upon cellular stress. Due to the heterogeneity of the microenvironment in cells, the same glycoprotein may play specific regulatory roles in different cellular compartments during cellular stress, but this spatial study has rarely been explored.

**Figure 5.**
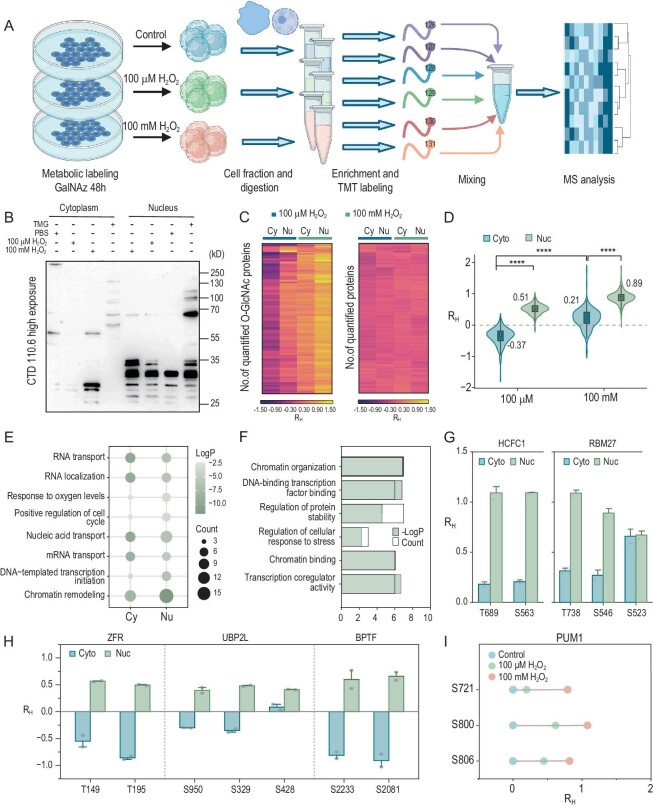
Quantitative proteomics analysis revealed the distinct spatial O-GlcNAcylation patterns between nucleus and cytoplasm during oxidative stress. (A) Experimental workflow for quantitative analysis of the O-GlcNAc proteins in the nucleus and cytoplasm. (B) Western blotting (detected by clone CTD110.6) for samples from different compartments with 100 μM H_2_O_2_ and 100 mM H_2_O_2_ treatment. CTD 110.6, O-GlcNAc antibody. TMG: Thiamet G, an O-GlcNAcase inhibitor. (C) Heat map of the quantified O-GlcNAc proteins (left) and the quantified proteins without enrichment (right). R_H_: log_2_ (H_2_O_2_ treatment/control) ratio, showing the normalized relative fold changes (compared with control) stimulated with H_2_O_2_. (D) Violin plot of R_H_ on O-GlcNAc proteins in different compartments under 100 μM H_2_O_2_ and 100 mM H_2_O_2_ treatment, respectively. In the boxplots, the center white point indicates the median value, and box limits indicate the first and third quartiles. The average R values (black) are indicated for each condition. Statistical significance was determined by student's t-test, two tailed. The significance levels are labeled ** (*P* < 0.01), *** (*P* < 0.001) and **** (*P* < 0.0001). (E) Comparison of the biological process terms for glycoproteins that have significantly changed in the nucleus and cytoplasm under 100 μM H_2_O_2_. (F) GO analysis of the O-GlcNAc proteins significantly up-regulated in the nucleus and significantly down-regulated in the cytoplasm under 100 μM H_2_O_2_. (G and H) Examples of O-GlcNAc proteins with site-specific quantification in the nucleus and the cytoplasm under 100 μM H_2_O_2_ (G) and 100 mM H_2_O_2_ (H). Error bars represent mean ± s.d. from two independent experiments. (I) The R_H_ of different O-GlcNAc sites in PUM1 under control, 100 μM H_2_O_2_ and 100 mM H_2_O_2_ treatment, respectively.

Here, we employed the HG-TCs and TMT labeling strategy to investigate the dynamics of O-GlcNAc proteins in the nucleus/cytoplasm upon oxidative stress (Fig. 5A). HeLa cells were treated with hydrogen peroxide (H_2_O_2_) as a model of acute oxidative stress. In brief, HeLa cells were cultured with GalNAz for 48 h and then treated with PBS, 100 μM H_2_O_2_ and 100 mM H_2_O_2_ for 1 h, respectively. We chose the concentrations of 100 μM and 100 mM to investigate the effect of oxidative stress at different doses [39]. Then, cells were harvested and lysed, and the nucleus and cytoplasm fractions were separated and collected. Next, HG-TC was performed simultaneously on six samples (including three nuclear samples and three cytoplasmic samples), which were then labeled with six-plex TMT reagents and mixed. The mixture was purified for LC-MS/MS analysis. To preliminarily investigate the status of O-GlcNAcylation under oxidative stress, we performed western blotting of samples treated with PBS or H_2_O_2_ (without metabolic labeling) using CTD110.6 antibody (Fig. 5B and Fig. S18A). The result showed that the O-GlcNAcylation levels in both the nucleus and cytoplasm changed dramatically after H_2_O_2_ treatment. In addition, six unenriched samples were labeled with six-plex TMT reagents and sent for LC-MS/MS analysis to reflect the dynamics of non-modified proteins (Fig. S18B) [41].

To better understand the O-GlcNAcylation dynamics under oxidative stress, we defined the log_2_ (H_2_O_2_ treatment/control) ratio as R_H_ to evaluate the relative changes of protein or peptide after H_2_O_2_ treatment. In total, we quantified 778 O-GlcNAc peptides corresponding to 255 glycoproteins from two replicates (Tables S12 and S13). The reproducibility across replicates was evaluated, and the correlations were reasonably high (Fig. S19). We selected 176 O-GlcNAc proteins localized in the nucleus/cytoplasm in UniProt that were quantifiable across all replicates for subsequent analysis (Table S14). Then, by analyzing the R_H_ values of proteins from samples without enrichment, we found that the variation of O-GlcNAc proteome was more significant than proteome expression (Fig. 5C). This result revealed that O-GlcNAc proteome is much more sensitive than global proteome under oxidative stress, so the observed changes may be driven more by the O-GlcNAcylation states of the proteins than by global perturbations.

Quantitative proteomic analysis revealed distinct spatial O-GlcNAcylation patterns during oxidative stress. The violin plot showed that O-GlcNAc proteins were up-regulated in the nucleus and down-regulated in the cytoplasm under a 100 μM H_2_O_2_ condition (Fig. 5D). This tendency was consistent with the western blotting result, showing the reliability and authenticity of the data. We compared the biological process terms for significantly regulated (>1.5-fold change) glycoproteins in the nucleus and cytoplasm. In the cytoplasm, glycoproteins involved in processes such as RNA transport, RNA localization and nucleic acid transport were enriched. In the nucleus, glycoproteins involved in transcription and chromatin remodeling were highly over-represented (Fig. 5E and Fig. S20A). Previous studies have shown that increased O-GlcNAcylation is protective in acute stress, particularly in cardiac cells [38]. Previous research has shown that reducing cellular O-GlcNAc levels produces deleterious effects [39]. Seventeen O-GlcNAc proteins significantly up-regulated in the nucleus and down-regulated in the cytoplasm were closely related to transcription, chromatin organization and protein stability regulation (Fig. 5F and Fig. S20B). This indicated that transcription-related O-GlcNAc proteins accumulate in the nucleus and disassociate in the cytoplasm to protect the cell. Given previous reports that O-GlcNAc proteins exhibit dose-dependent responses to stressors like UVB light or sodium arsenite, we investigated the effect of an H_2_O_2_ dose on O-GlcNAcylation [39]. However, O-GlcNAc proteins showed an increase in both the nucleus and cytoplasm under a 100 mM H_2_O_2_ condition (Fig. 5D). This tendency was consistent with results previously reported by Zachara's group at the global level [40].

The site-specific and spatial quantification of glycoproteins also revealed independent changes of O-GlcNAc sites in the nucleus and cytoplasm (Fig. 5G and H; Table S10). For some sites on the same protein, changes were very similar. For example, on protein ZFR, both T149 and T195 displayed up-regulation in the nucleus and down-regulation in the cytoplasm upon 100 μM H_2_O_2_ induction (Fig. 5H). Conversely, for the protein RBM27, glycosites T738 and S546 showed more distinct changes in the nucleus than in the cytoplasm, while T523 showed similar trends in both compartments (Fig. 5G). According to previous reports, cellular injury induces widespread changes in transcriptional patterns, many of which require adenosine triphosphate (ATP)-dependent chromatin remodelers [42]. Glycosites S2233 and S2081 on protein BPTF, a component of the switch defective/sucrose non-fermentable (SWI/SNF) chromatin remodeling complex, showed a similar trend under 100 μM H_2_O_2_ (Fig. 5H). Moreover, many proteins involved in cellular stress response were observed, such as stress granule (SG) components. We identified three reported glycosites (S721, S800 and S806) on the SG protein PUM1, and found that S800 and S806 were more sensitive to low concentration of H_2_O_2_ than S721 (Fig. 5I). In conclusion, variations of O-GlcNAc proteins and sites across different compartments and doses provided an interesting insight into the complex and reciprocal crosstalk between O-GlcNAcylation and cellular perturbation.

## CONCLUSION

In this work, we established a rapid and large-scale HexNAc-containing glycopeptide or glycan enrichment strategy based on TC-resin (HG-TCs) and applied it to the comprehensive glycoproteome profiling and spatial-resolved investigation of O-GlcNAcylation under oxidative stress. The success of HG-TCs can be partially attributed to the convenient and practical TC-resin. However, the potential applications of this material are extensive. Since TC-resin can capture any peptide with an azide group, it can be combined with enzymatic labeling to manipulate serum or tissue samples and not be limited to cells [43]. Additionally, it can be used to enrich newly synthesized proteins or peptides containing specific amino acids [44]. More importantly, HG-TCs can enrich and identify various types of glycosylation simultaneously, which can improve data comparability and reliability, save time/experimental resources, increase efficiency and reduce the amount of sample, especially for precious samples. In various biological research fields, simultaneous study of multiple types of glycosylation is necessary. One prominent example is cancer research. Tumor cells display a wide range of glycosylation alterations: not only in comparison to non-transformed counterparts but also in glycosylation patterns during the different stages of the tumor [45]. There are two principal mechanisms underlying the tumor-associated alterations of carbohydrate structure [46]. The incomplete synthesis process, occurring more often in the early stages of cancer, leads to the biosynthesis of truncated structures, such as the expression of short-chain O-GalNAc glycans (Tn and Sialyl-Tn) in gastrointestinal and breast cancers [47,48]. Conversely, the neo-synthesis process, commonly observed in advanced stages of cancer, leads to the *de novo* synthesis of neoantigens like Sialyl-Lewis A (SLe^a^) and X (SLe^x^), and complex branched N-glycans [49]. Therefore, HG-TCs are very conducive to the study of such a highly dynamic and complex carbohydrate system, allowing not only the mapping of multiple glycosylations but also the simultaneous monitoring of multiple glycosylation alterations.

However, this method has some limitations. Although we refer to previous studies to assign O-GlcNAc and Tn-antigen based on subcellular localization [13], HG-TCs still have limitations in distinguishing them based on the mass of modification. In addition, since we used only HCD for tandem fragmentation, the fragmentation information obtained was not comprehensive enough for other mucin-type glycopeptides, thus making it difficult to retrieve information about it. The Click-iG platform has also enabled various types of glycosylation profiling at the level of intact glycopeptides, thus providing information on both the glycosites and the glycan compositions [13]. However, it is demanding on MS/MS fragmentation type, glycan databases and analytical software. HG-TCs simplifies the process through the convenient enrichment procedure, the universal MS method and the conventional analytical program, thereby alleviating the practical difficulties associated with highly technical requirements. Therefore, the Click-iG platform can obtain more comprehensive information, and HG-TCs can be a versatile and widely applicable tool for glycoproteomics profiling in scientific and clinical research. By balancing these approaches, researchers can leverage the strengths of both strategies to achieve comprehensive and efficient glycopeptide analysis in the future.

## METHODS

A detailed description is included in the supplementary data.

## Supplementary Material

nwae341_Supplemental_Files

## Data Availability

The RAW MS data as well as the original search results have been deposited in the ProteomeXchange Consortium via the iProX repository, with the data set identifiers PXD048703, PXD048484 and PXD48827.
