# Supplementary material for: Rapid and large-scale glycopeptide enrichment strategy based on chemical ligation
Source: Natl Sci Rev. 2024 Sep 27;11(11):nwae341. doi: 10.1093/nsr/nwae341 (PMC11556338; doi:10.1093/nsr/nwae341)
Supplement: nwae341_Supplemental_Files [file nwae341_supplemental_files.zip › 484-Supplemetary information.pdf]

Supplementary Information

Rapid and large-scale glycopeptides enrichment strategy based on chemical ligation

Yingying Xiong[1], Zhuoer Lu[1], Yuyin Shao[2], Peiyi Meng[1], Guoli Wang[2], Xinwen Zhou[2], Jun Yao[2], Hui-min Bao*[1], Haojie Lu*[1,2,3]

[1]Department of Chemistry and Liver Cancer Institute, Zhongshan Hospital, Fudan University, Shanghai 200032, China

[2]Institutes of Biomedical Sciences and NHC Key Laboratory of Glycoconjugates Research, Fudan University, Shanghai 200032, China

Correspondence: baohm@fudan.edu.cn; luhaojie@fudan.edu.cn

Supplementary Text

Cell culture and metabolic labeling

HeLa cells (from Cell Resource Center of the Shanghai Institute for Biology Science, Chinese Academy of Science) were grown in high glucose Dulbecco's Modified Eagle's Medium (DMEM, Sigma-Aldrich) containing 10% fetal bovine serum (FBS, Gibco) in a humidified incubator with 5.0% CO_2 at 37 °C. The cell confluency was monitored regularly. When HeLa cells were grown to 70%, 500 μM GalNAz (Glycogene Inc, #MP005) was added and the cells were further cultured for 48 h. After incubation for 24 h, 10 μM TMG (Sigma-Aldrich, #SML0244) was added, and the cells were further cultured for 24 h. For the experiment with the hydrogen peroxide (H_2O_2) treatment, cells were cultured with 500 μM GalNAz for 48 h. Then the cells were treated with PBS (pH 7.2), 100 μM H_2O_2, and 100 mM H_2O_2 for 1 h, respectively. The cells were harvested by centrifugation at 1000 g for 5 min at 4 °C. Then cells were washed with ice-cold PBS (pH=7.2) three times before protein extraction.

Nucleus/cytoplasm/membrane protein extraction and digestion

Cell fractionation was performed following previous publications [1]. The cells were first resuspended into AS buffer (30 mM HEPES, 15 mM NaCl, 350 mM sucrose, 20 μM TMG, 1 tablet/10 mL EDTA-free protease inhibitor (Roche), pH=7.4) containing 0.015% digitonin (Sigma-Aldrich) and 0.5% Tween-20 (Sigma-Aldrich). The cell suspension was gently triturated 5 times, incubated on ice for 35 min, and centrifuged at 500 g for 3 min. The supernatant was recovered and transferred to a clean tube labeled as the cytoplasmic fraction. Then cell pellets were washed with 1 mL of AS buffer and resuspended in AG buffer (30 mM HEPES, pH=7.4, 15 mM NaCl, 20% glycerol, 20 μM TMG, 1 tablet/10 mL EDTA-free protease inhibitor) containing 1% dodecyl maltoside. The operation is the same as above. The supernatant was recovered as membrane fraction and cell pellets were washed with 500 μl of AG buffer and resuspended in nucleus isolation buffer (30 mM HEPES, 500 mM NaCl, 20 μM TMG, 1 tablet/10 mL EDTA-free protease inhibitor, pH=7.4) containing 0.2% SDS. Samples were incubated on ice for 1h and sonicated for 3 min. The supernatant was collected as a nuclear fraction by centrifugation at 18,000 g for 10 min at 4 °C. After heating for 15 min at 75 °C, all samples were digested overnight at 37 °C by trypsin at a 1:50 w/w ratio and stored at -80°C until the next step.

Synthesis of alkyne/azide-peptide

The alkyne/azide-peptides were synthesized by ChinaPeptides Co., Ltd., by adding amino acids in the reactor according to the known sequence (from the carboxyl-terminal to the amino-terminal), and then reacting, synthesizing, and purifying to obtain the final product. The procedures are as follows: (1) Put Rink Amide-MBHA Resin into a reaction tube, add dichloromethane (DCM, 15 mL/g), shake for 30 min, filter out the solvent and rinse. (2) Add a 3 mol of Fmoc-protected amino acid (the first amino acid at the C-terminal, dissolved in Dimethylformamide (DMF)), then add 10 mol of diisopropylethylamine (DIEA), and shake for 60 min. Blocked with pyridine and acetic anhydride. (3) Wash the Resin with the following: DMF (10 mL/g) twice, methanol (10 mL/g) twice, DMF (10 mL/g) twice. (4) Add a 3 mol of Fmoc-protected amino acid, a 3 mol of 1-hydroxybenzotrichloroazolium tetramethyl-hexafluorophosphate (HBTU), and a 10 mol of DIEA, and shake for 45 min. (5) Repeat steps 3 to 5 with the addition of the different amino acids in the sequence (from right to left). (6) The resin is blown dry with nitrogen and reacted for 180 min in the following cutting solution (TFA 95%, water 2%, ethylenedithiol (EDT) 2%, triisopropylsilane (TIS) 1%), then the resin is filtered off. (7) Ether is added to the filtrate to precipitate the crude product. The supernatant is removed by centrifugation, and the precipitate is washed with ether and evaporated at room temperature. (8) The crude product was dissolved with H_2O/ACN and purified by using C18 reversed-phase chromatography system (Wavelength:220 nm; Flow Rate:15 mL/min; Inj.Vol: 20 mL Column; Temp: 25 ℃; Buffer A: 0.1% TFA in water; Buffer B: 0.1% TFA in Acetonitrile), and the target peak solution was collected and lyophilized.

Synthesis of TC-resin

The synthesized alkyne-peptide (GLAGLLARG-alkyne) containing one tryptic cleavage site was immobilized on the AminoLink aldehyde-functionalized resin (Thermo Fisher Scientific, #20505) with the following protocol.

First, the resin was washed with PBS three times and then incubated with alkyne-peptide in the presence of 50 mM Sodium cyanoborohydride ($NaBH_3CN$, Aladdin, #S107168) at 37 °C for 10 h. The flow-through were collected for MALDI-TOF-MS analysis. Next, the resin was blocked by incubating with 1 M Tris buffer (pH=7.4) and 50 mM $NaBH_3CN$ at 37 °C for 2 h. After washing with Tris-HCl buffer and 1 M NaCl three times, the resin was suspended in PBS and stored at 4 °C until needed. To verify whether the alkyne peptide was successfully attached to the resin, we incubated the alkyne peptide and aldehyde resin with or without $NaBH_3CN$ and detected the flow-through by MALDI-TOF-MS.

Characterization of TC-resin

Scanning electron microscopy (SEM) was performed on a Hitachi FlexSEM1000 scanning electron microscope at an accelerating voltage of 20 kV. Samples dispersed to the appropriate concentration were cast onto glass plates at RT and then sputter-plated with gold. Fourier transform infrared spectra (FT-IR) were conducted on a Magna-550 (Nicolet, USA) spectrometer. Spectra were scanned over the range of 400-4000 cm^{-1}. The dried samples were first mixed with KBr, after which the mixtures were compressed into pellets.

Capture of the standard O-GlcNAz peptides

The standard O-GlcNAz peptides SGP (PGGSTPVSS#$^{(+GlcNAz)}$ ANMM) (aq, 1 μg/μL) was added into a tube of 49 μL of 100 mM PBS (pH=7.2) containing 5 μL alkyne-TC-resin. Then, 1 mM $CuSO_4$, 5 mM BTTP (Click Chemistry Tools, #CCT-1414), and freshly prepared 15 mM sodium L-ascorbate (Sigma-Aldrich, Cat#A7631) were added to the solution. The click reaction was completed by incubating for 1 h at room temperature with vigorous shaking. The solution was centrifuged by 500 g for 2 min and the supernatant was transferred to a clean tube. Then resin was washed with 500 μL of PBS three times and then resuspended in 50 mM ammonium bicarbonate, and the enriched glycopeptides were eluted from the resin by trypsin (Hualishi Tech. Ltd., Cat#HLS TRY001C) cleavage at 37 °C for 4 h. The resulting peptides and click supernatant were collected and desalted using C18 ZipTips (Millipore, #ZTC18S096), freeze-dried, and stored at -80 °C for MALDI-TOF-MS analysis.

Stability analysis of the azide-peptides and removal of trypsin activity

The azide peptide (GLAGLLARG-N_3) (aq, 1 μg/μL) was added into three tubes of 19 μL PBS buffer respectively. Then, these tubes were incubated under these conditions: (i) at room temperature for 30 min; (ii) with 10 mM DTT at 56 °C for 30 min; (iii) at 75 °C for 30 min. The steps for the O-GlcNAz peptide were the same as above. For the removal of tryptic activity in cell lysate, 1 μL of linker peptides (GLAGLLARG-alkyne) (aq, 1 μg/μL) was added into three tubes of 19 μL PBS buffer respectively. Then, these tubes were incubated under these conditions: (i) at 37 °C for 4 h; (ii) with trypsin-treated lysates (trypsin digestion products) at 37 °C for 4 h; (iii) trypsin-treated ly-sates were heated at 75 °C for 20 min, then incubated with linker peptides at 37 °C for 4 h. After incubation, these solutions were desalted using C18 ZipTips, freeze-dried and stored at -80 °C for MALDI-TOF-MS analysis.

Glycopeptide capture and release

Glycopeptides from cell samples were captured by alkyne-TC-resin through the copper(I)-catalyzed azide-alkyne cycloaddition (CuAAC) reaction. Briefly, an amount of 20 μL of synthesized alkyne-TC-resin, 1 mM $CuSO_4$, and 5 mM BTTP were added to the lysate containing 500 μg peptides. After thorough mixing, freshly prepared 15 mM sodium L-ascorbate was added to initiate the reaction. The CuAAC reactions were performed for 1 h at room temperature with vigorous shaking. The resins then were washed sequentially with 100 column volumes of the following four buffers: (i) 0.75 % SDS, 250 mM NaCl, 5 mM EDTA, 100 mM Tris (pH=8.0); (ii) 8 M urea; (iii) 50% acetonitrile; (iv) 50 mM ammonium bicarbonate. The entire wash process took about 1 h. After the wash step, the resin was resuspended in 50 mM ammonium bicarbonate, and the enriched glycopeptides were eluted from the resin by trypsin cleavage at 37 °C for 4 h. The resulting peptides were desalted using C18 ZipTips, freeze-dried, and stored at -80 °C for future use. For the N-glycopeptide and O-GlcNAc peptides simultaneous enrichment, samples were not pre-treated with PNGase F before enrichment. After the CuAAC and wash step, the resin was resuspended in 25 mM ammonium bicarbonate in heavy-oxygen water ($H_2{}^{18}O$), and

then treated with PNGase F to a ratio of 1 μg: 1 U and incubated at 37 °C for 3h. The resulting peptides were desalted using C18 ZipTips, freeze-dried, and stored at -80 °C for future use.

N-glycan purification using sterilized cotton wool

The glycopeptides were desalted using C18 ZipTips, and the wash buffer and flow-through were collected for cotton enrichment. The cotton enrichment was performed following previous publications [2].

TMT labeling

The TMT labeling approach was adopted from the reported protocol with slight modifications [3]. For the analysis of O-GlcNAc proteins in response to oxidative stress, three cytoplasmic samples from different treatments (control, 100 μM H_2O_2, 100 mM H_2O_2) were labeled with the three channels (126, 127, and 128) of the TMT six-plex reagents (Thermo Fisher Scientific, #90066), and three samples of the nuclear fractions were labeled with the other three channels (129, 130, and 131), respectively. To exclude differences in protein abundance under different stimulation conditions, six unenriched samples were also labeled with 6-plex TMT reagents as described above. Briefly, the lyophilized peptides were resuspended in 33 μL of 100 mM HEPES, pH=8.5 and 10 μL ACN. Each tube of the TMT labeling reagent was dissolved in 41 μL ACN and then 10 μL of the solution was transferred to the designated sample. The labeling was performed for 1 h at room temperature, and the reaction was quenched by adding 4 μL of 1 M Tris (pH=8). The samples were mixed, desalted using C18 ZipTips, freeze-dried, and stored at -80 °C for future use.

LC-MS/MS and MALDI-TOF-MS analysis

The peptides were subjected to NSI source followed by tandem mass spectrometry in Orbitrap Exploris 480 (Thermo Fisher Scientific, MA, USA) coupled online to the UPLC. In brief, peptide samples were dissolved in solvent A (0.1% formic acid), and 2 μg of each sample was analyzed. Then samples were directly loaded onto a reversed-phase analytical column (Acclaim PepMap C18, 75 μm x 50 cm). The gradient was comprised of an increase from 2% to 35% solvent B (0.1% formic acid in 98% acetonitrile) over 75 min, 35% to 55% in 9 min, and climbing to 80% in 1min then holding at 80% for the last 5 min, all at a constant flow rate of 300 nl/min on an EASY-nLC 1200 UPLC system. The peptides were subjected to NSI source followed by tandem mass spectrometry in Orbitrap Exploris 480 (Thermo Fisher Scientific, MA, USA) coupled online to the UPLC. The spray voltage was set to 2.3 kV, the funnel RF level at 50, and the heated capillary temperature at 320 °C. For DDA experiments full MS resolutions were set to 60,000 at m/z 200 and the full MS AGC target was 300% with injection time of 50 ms. The mass range was set to 350-1800 m/z. AGC target value for fragment spectrum was set at 100% with a resolution of 15,000 and injection times of 50 ms and Top20. The intensity threshold was kept at 2E4. Isolation width was set at 1.6 m/z. The fragmentation of precursor ions was performed by HCD with a normalized collision energy of 28%.

The standard azido peptide analysis and N-glycan analysis were performed on rapifleX® (Bruker Daltonics, MA, USA) in the positive reflection mode with a wavelength of 355 nm, a repetition rate of 400 Hz, and an acceleration voltage of 20 kV. For the peptides sample, the matrix solution was 5 μg/μL α-cyano-4-hydroxycinnamic acid (CHCA) dissolved in 50% ACN/0.1% TFA; for the N-glycan sample, the matrix solution was 2,5-dihydroxybenzoic acid (DHB). Samples and matrix solution were dropped onto the MALDI plate for MS analysis.

Statistical Analysis

For the O-GlcNAc/O-GalNAc peptide identification, the raw LC-MS data were searched against the human database (Homo sapiens, 20,375 protein entries downloaded from UniProt in November 2022) using Byonic (v.4.6.1, Protein Metrics, San Carlos, CA) against the respective protein sequence and decoys. The protease was fully specified as trypsin with two maximum missed cleavages. The following parameters were employed during the search: 20 ppm precursor mass tolerance; 0.05 Da for the fragment; scan type: HCD; variable modifications: oxidation of methionine (+15.9949 Da), protein N-terminal acetylation (+42.010 Da), O-GlcNAc/O-GalNAc (HexNAc (1)153.065057 on S or T). The false discovery rates (FDRs) of glycopeptides were filtered to less than 1%. To ensure the high quality of the glycopeptide identification, additional filters including Byonic score > 150,

PEP 2D < 0.05, and FDR 2D < 0.01 were implemented. There is also an additional filter: only glycopeptides with specific oxonium ions at m/z 357.15 in the MS/MS spectra were confirmed to O-HexNAc glycopeptides. All selected MS/MS spectra of O-GlcNAc glycopeptides were manually confirmed. To ensure the reliability of the data, we assign O-GlcNAc and O-GalNAc (Tn antigen) manually based on the subcellular localization. The O-HexNAc modifications occurring on the cytoplasmic side (including the modified proteins localized in the nucleus, cytoplasmic, mitochondrial, and the cytoplasmic part of transmembrane proteins) were assigned as O-GlcNAc, and those occurring in the lumen and extracellular part were assigned as Tn antigen [4].

For the N-glycosite identification, the raw LC-MS data were searched with 10 ppm mass tolerance for the precursor and 0.02 Da for the fragment using PEAKS Online Xpro v.1.6 (Bioinformatics Solutions Inc.). Trypsin was selected as the enzyme with a specific digestion mode, and no more than two missed cleavages were allowed. Oxidation of methionine (+15.9949 Da), protein N-terminal acetylation (+42.0106 Da), and ^{18}O tag of Asn (+2.9883 Da) were set as variable modifications. A 1% FDR was used as a filter at both PSM and protein levels. Additionally, all N-glycosylation sites were required to contain the motif N-X-[S/T], where X is any amino acid except proline.

For the O-GlcNAc peptide quantification, the raw LC-MS data were searched against the human database (Homo sapiens, 20,375 protein entries downloaded from UniProt in November 2022) with 10 ppm mass tolerance for the precursor and 0.02 Da for the fragment using PEAKS Online Xpro v.1.6 (Bioinformatics Solutions Inc.). Trypsin was selected as the enzyme with a specific digestion mode, and no more than two missed cleavages were allowed. Oxidation of methionine (+15.9949 Da), and O-GlcNAc-TC-TMT (+585.3074 Da on S or T) were set as variable modifications. A 1% FDR was used as a filter at both PSM and protein levels. TMT-6-plex was set as the quantification method with a reporter ion type of MS2. For all other parameters, the default settings were used. For subsequent statistical analysis, the intensities of the reporter ions in H_2O_2 treatment channels were used to calculate the ratios against the control channels (126/127, 126/128, 129/130, 129/131) for glycopeptide quantification upon oxidative stress, and the $\log_2$ (H_2O_2 treatment /control) ratio was defined as R_H. For every unique glycopeptide, the intensity of the reporter ion is the sum up of all intensities of the reporter ions from the same peptides quantified here; For every glycoprotein, the intensity of the reporter ion is the sum up of the intensities from all the glycopeptides belonging to this protein. Furthermore, the peptide R_H ratio was the average of ratios from the replicate experiments, and the protein R_H ratio was the average of ratios from the replicate experiments. In addition, to exclude interference from contamination in nucleus/cytoplasm extraction, we removed proteins annotated with the keyword “membrane” or “secreted” in the UniProt.

For N-glycan search and identification, all MALDI-TOF-MS spectra were analyzed using flexAnalysis 4.0. The Peak Detection Algorithm was set to 'Snap', the Signal to Noise Threshold to 3, and isotope peaks were merged. All peak areas and signal-to-noise ratios were extracted from the peak list, providing quantitative information based on these areas. N-glycan identification was performed using glycoworkbench v2.1. The molecular weight search error was set to 50 ppm, and all the results were reviewed manually. The human N-glycans database was generated from the glygen database (https://glygen.org/).

Statistical analysis was performed using Excel and OriginLab. The statistical details can be found in the figure legends. Statistical significance was determined by the student’s t-test, two-tailed. The significance levels are labeled ** ($P < 0.01$), *** ($P < 0.001$), and **** ($P < 0.0001$). Error bars represent mean ± *s.d.* from all independent experiments.

Bioinformatic analysis

Protein subcellular location annotation information was obtained from UniProt (https://www.uniprot.org/). The GO analysis and protein-protein interaction networks were directly analyzed by using the metascape (https://metascape.org/) [5]. The motif analysis was performed by pLogo (https://plogo.uconn.edu/) [6]. The illustrations of HeLa cell, nuclear, and cytoplasm were generated by BioRender (https://www.biorender.com/). The list of human transcription factors was extracted from the Human Transcription Factors (http://humantfs.ccbr.utoronto.ca/) [7]. The information for reported O-GlcNAc proteins and sites were from the O-GlcNAcome database (https://www.oglcnac.mcw.edu/) and the O-GlcNAcAtlas database (https://oglcnac.org/) [8, 9].

Supplementary Figures

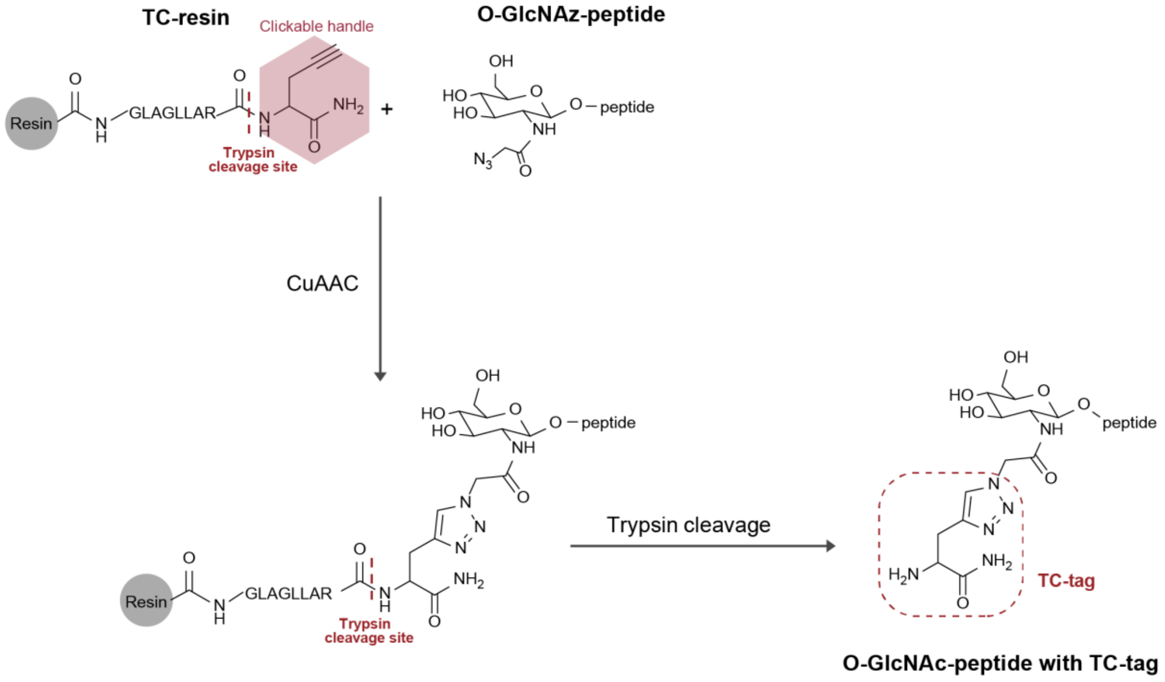

Fig. S1. The structural schematic of the process including the trypsin cleavage and the creation of the TC tag. TC-tag, the trypsin cleavage tag.

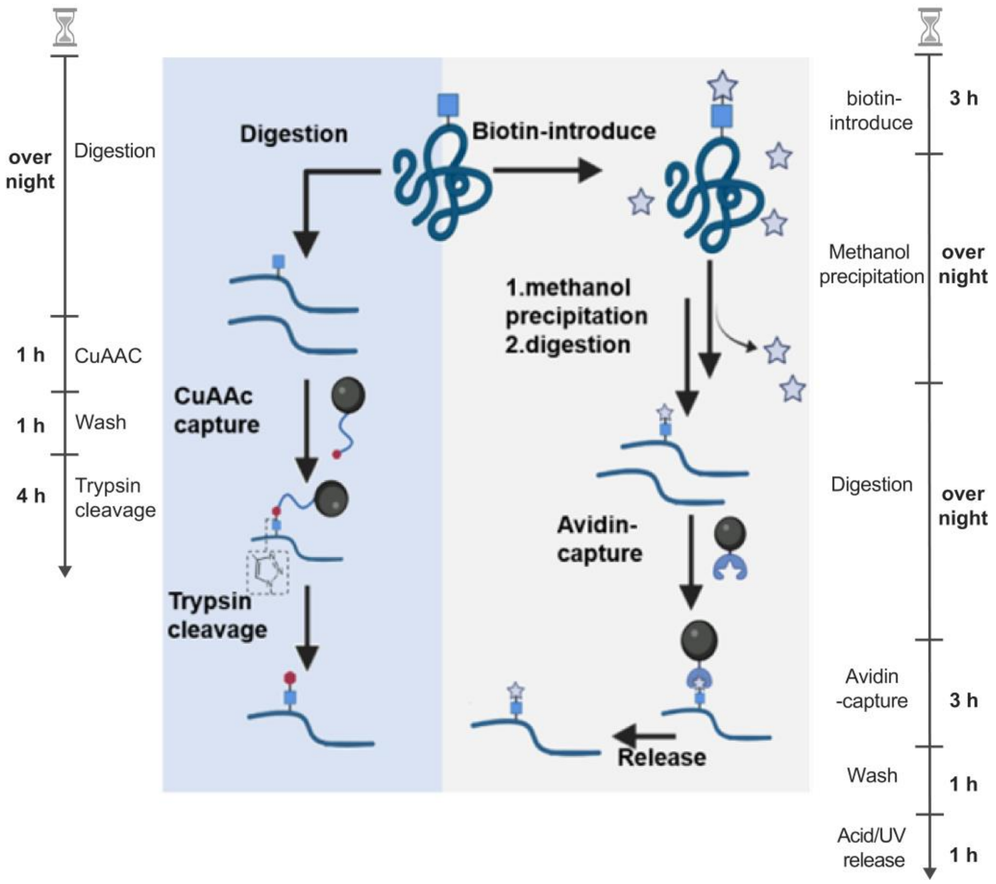

Fig. S2. Scheme of the HG-TCs and biotin-avidin workflows for O-GlcNAc proteome analysis.

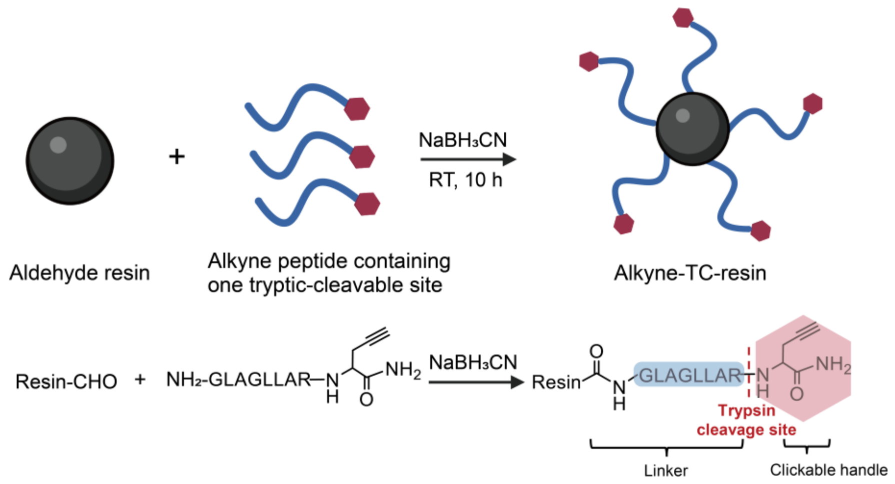

Fig. S3. Scheme of the preparation principle and preparation workflow of TC-resins. $NaBH_3CN$, sodium cyano-borohydride. RT, room temperature.

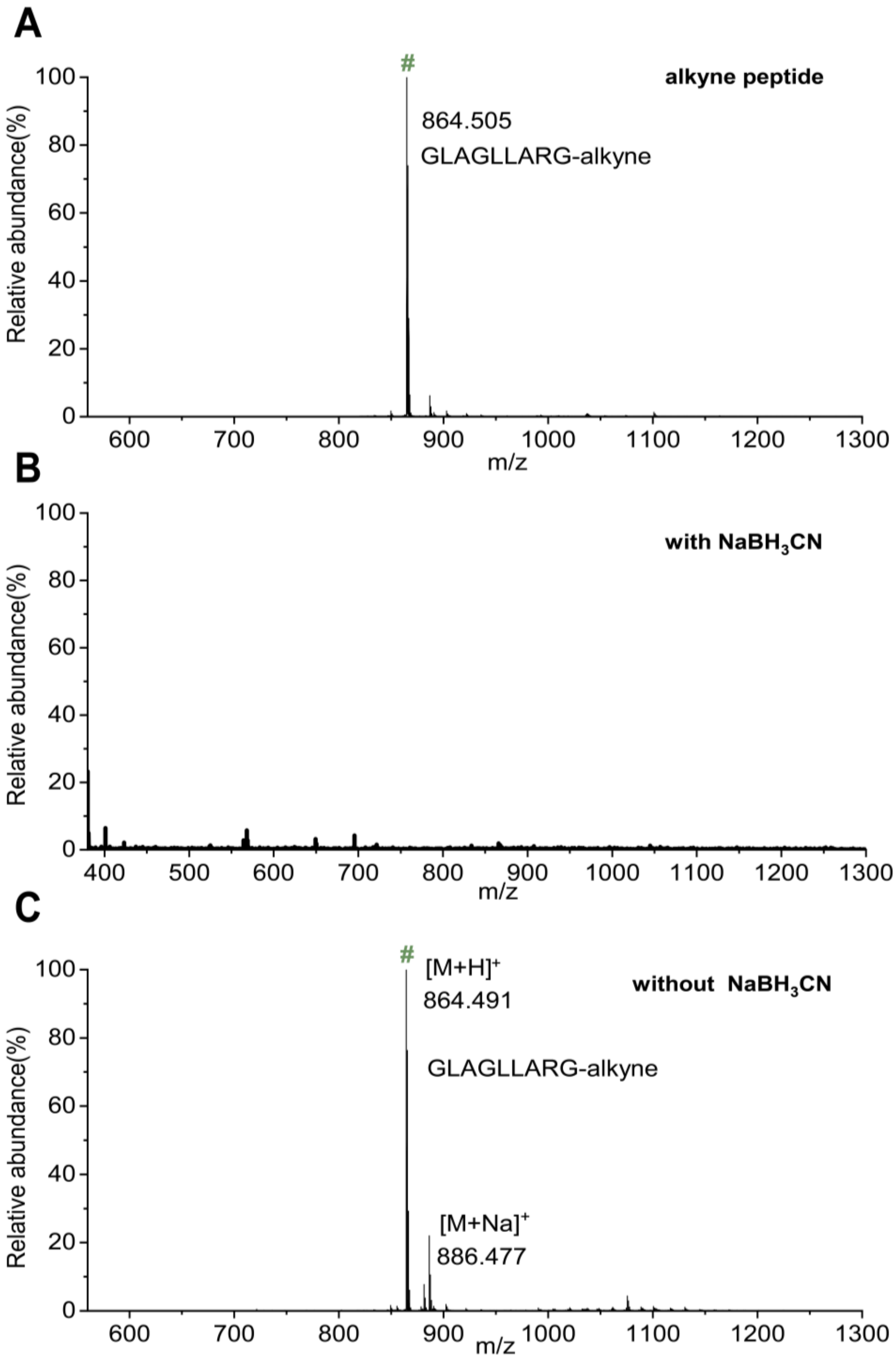

Fig. S4. The successful conjugation of alkyne peptides to resins. The MALDI-TOF-MS spectra of the alkyne peptide (A), the supernatant after the incubation of aldehyde resin and alkyne peptide with $NaBH_3CN$ (B) or without $NaBH_3CN$ (C).

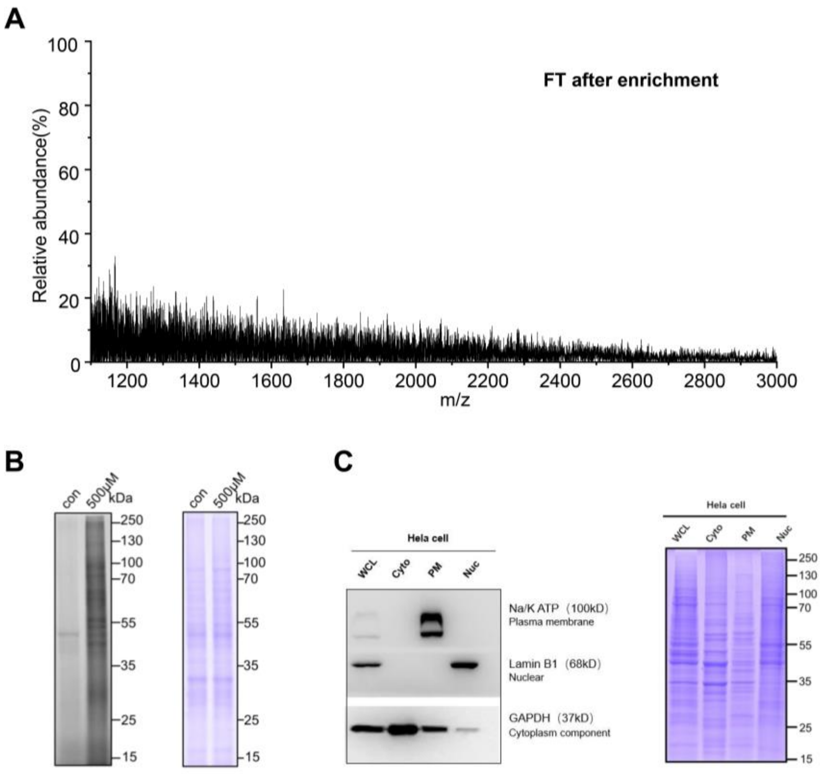

Fig. S5. Application of alkyne-TC-resin in profiling the O-GlcNAz peptide and the O-GlcNAc proteome from cell samples. (A) The MALDI-TOF-MS spectrum of the supernatant after the CuAAC reaction. (B) In-gel fluorescence imaging and coomassie blue staining of HeLa cell samples metabolically labeled by GalNAz and conjugated with alkyne-rhodamine via the CuAAC reaction. (C) Western blotting and coomassie blue staining of different cell fractions. WCL, whole cell lysate. Cyto, cytoplasm fraction. PM, plasma membrane fraction. Nuc, nuclear fraction.

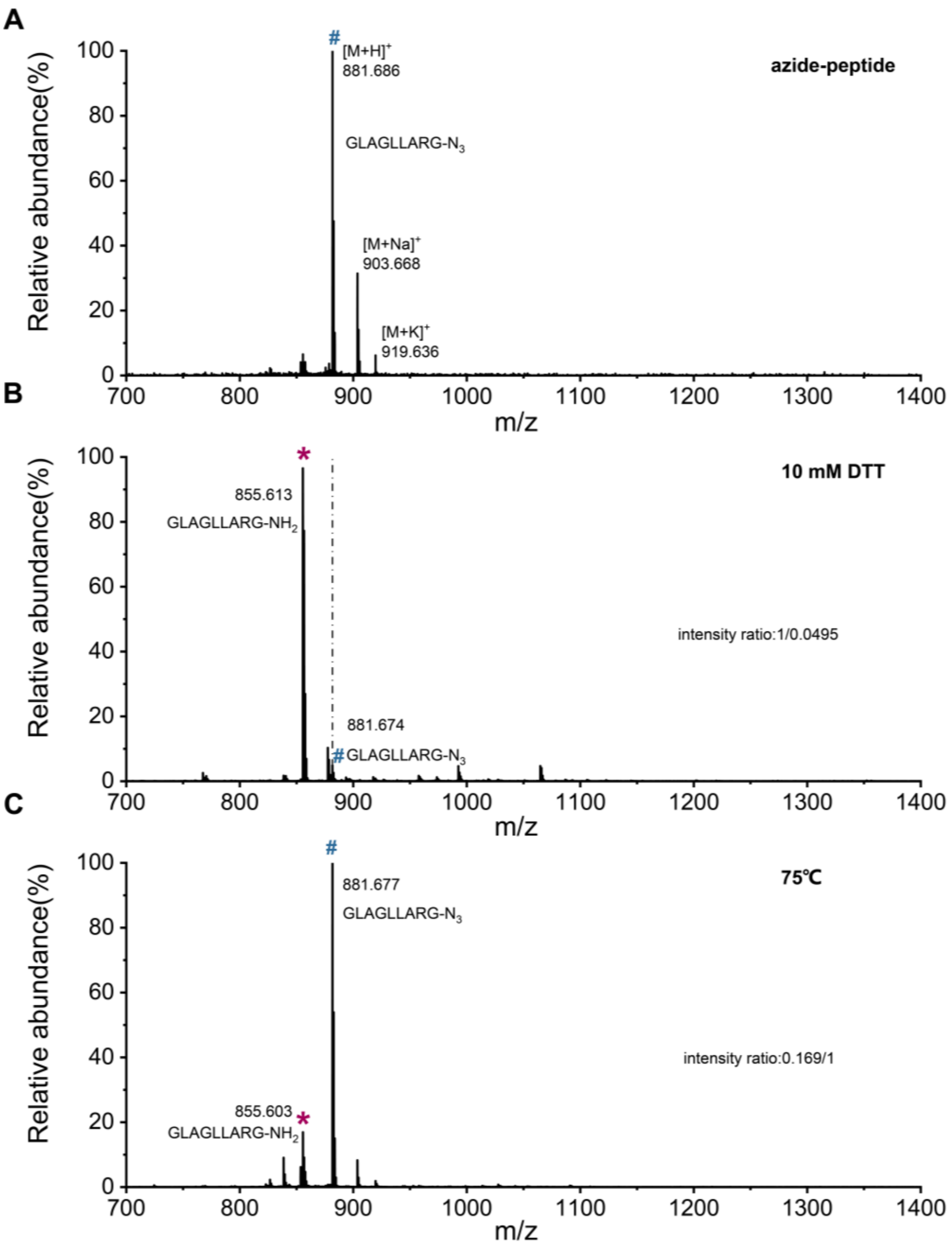

Fig. S6. The MALDI-TOF-MS spectra of the azide-peptide (A), the azide-peptide after incubation under 10 mM DTT for 30 min (B), or heating at 75 °C for 30 min (C).

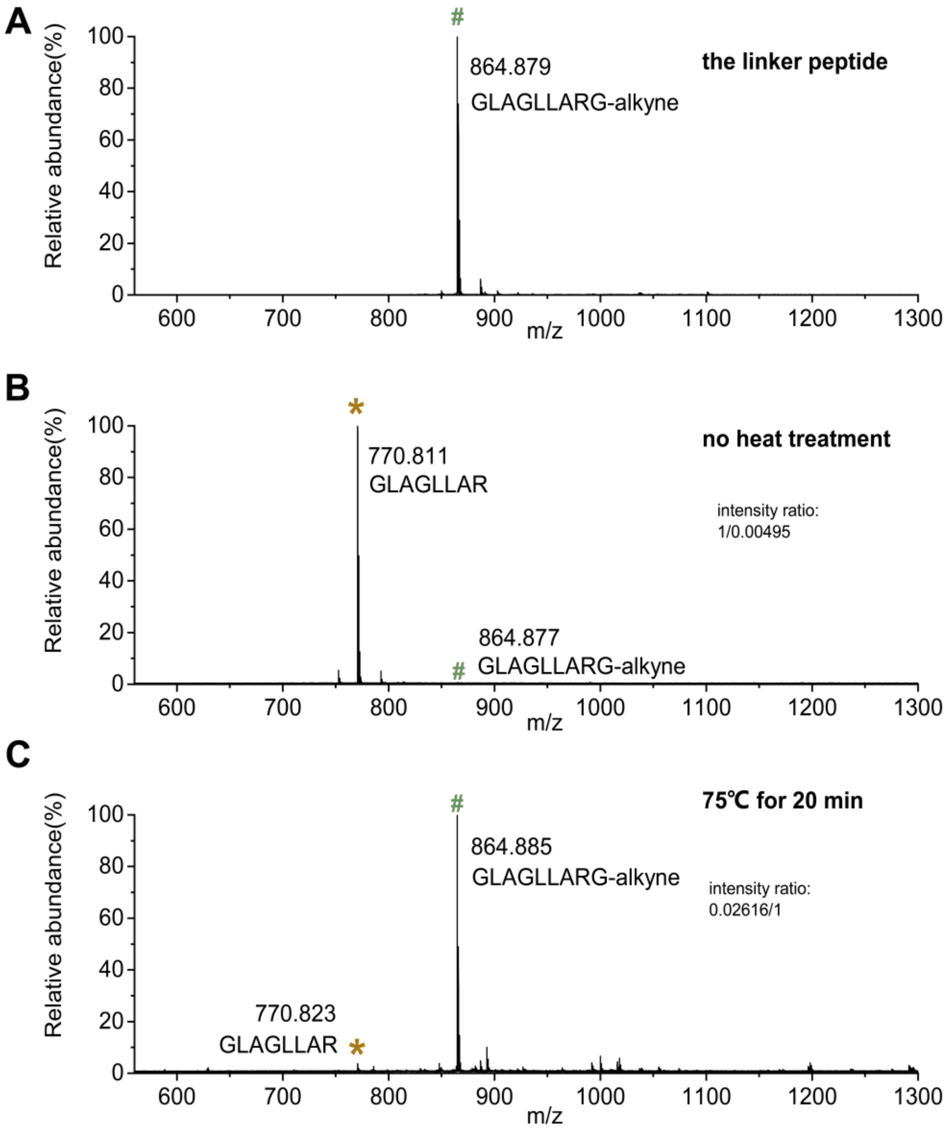

Fig. S7. The MALDI-TOF-MS spectra of the linker peptide (A), the trypsin-treated lysate incubated directly with the linker peptide at 37 °C for 4 h (B), and trypsin-treated lysate heated at 75 °C for 20 min, followed by incubation with the linker peptide at 37 °C for 4 h (C).

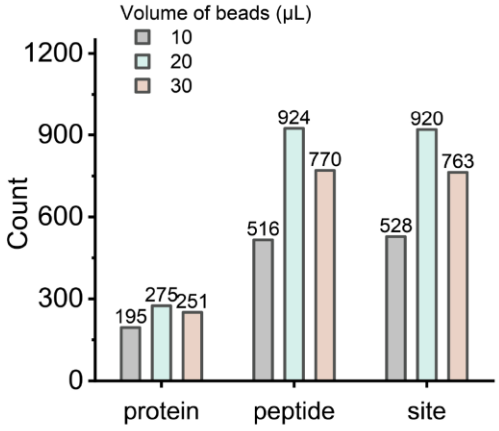

Fig. S8. Numbers of identified glycoproteins, glycopeptides, and glycosites using different amounts of alkyne-TC-resin (from 10, 20 to 30 μL) from 500 μg starting samples.

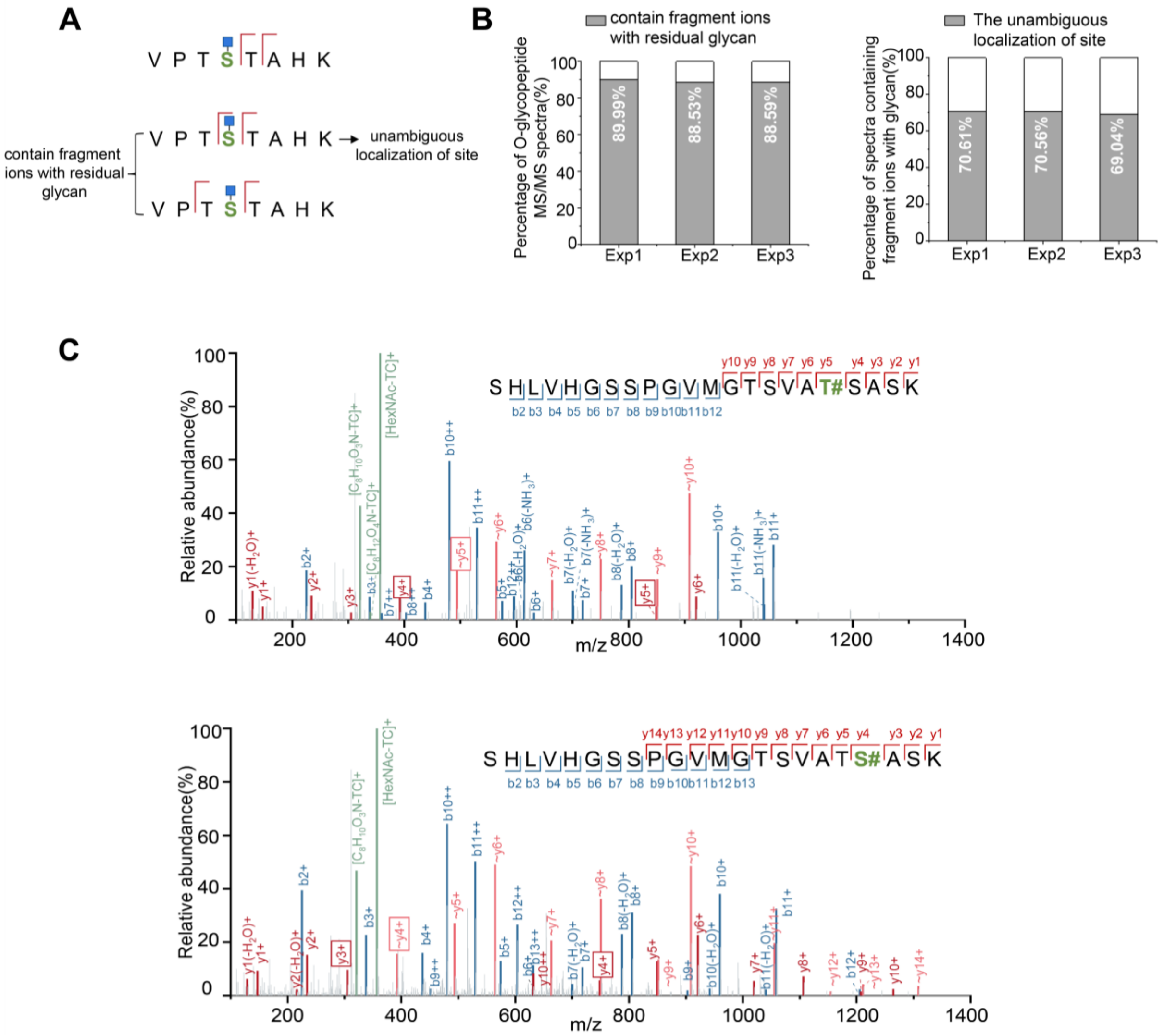

Fig. S9. Profiling of the O-GlcNAc proteome in the cell nucleus. (A) Schematic illustration of fragment ions containing residual glycan that facilitate site localization. (B) Percentage of MS/MS spectra contain fragment ions with residual glycan and the percentage of these spectra enabling unambiguous localization of site in individual experiment runs. (C) HCD MS/MS spectra of two glycopeptides on NUP214 with the same peptide sequence (Thr1055 and Ser1056 as the O-GlcNAc sites).

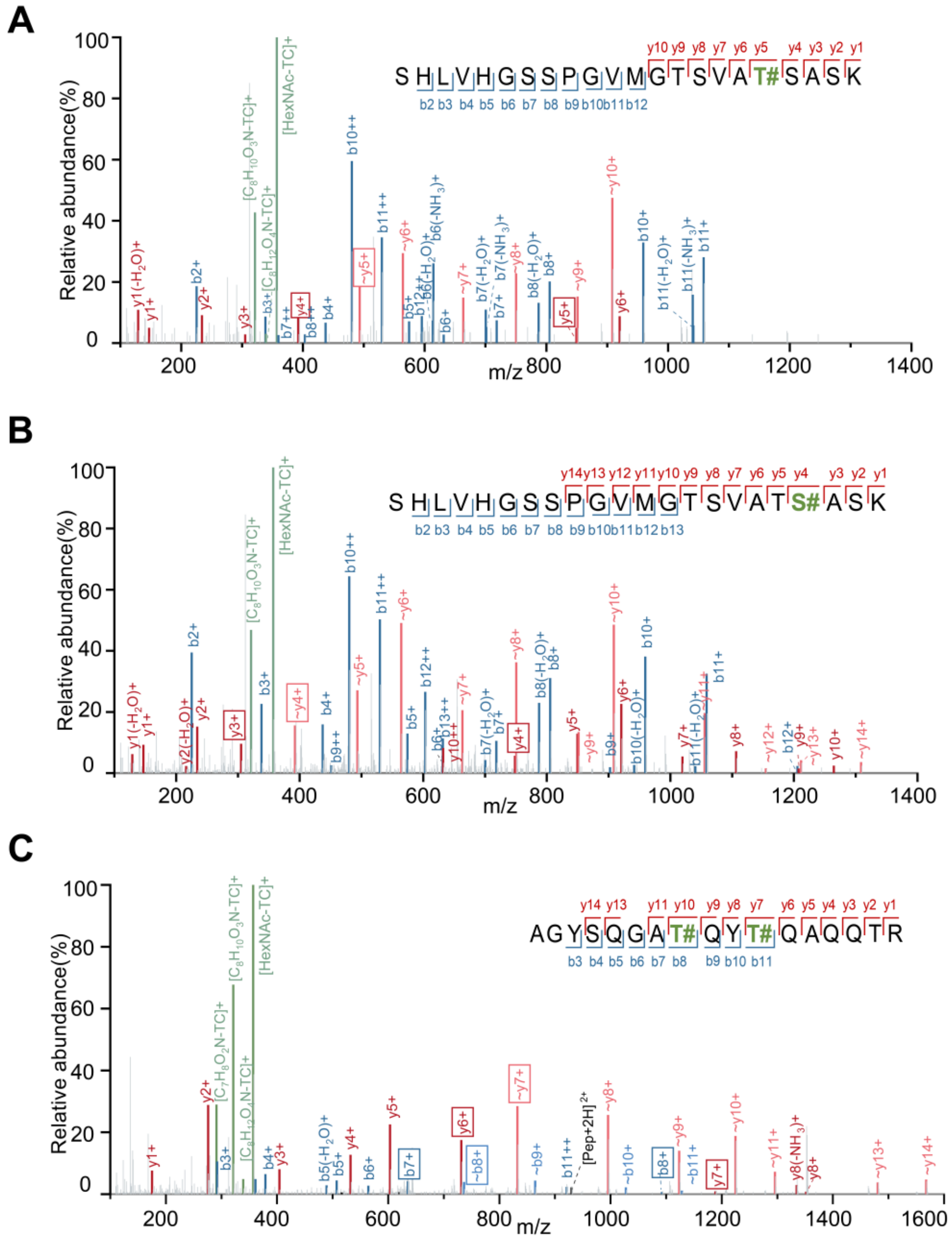

Fig. S10. Glycopeptides carried multiple O-GlcANc sites were identified with high sequence coverage. HCD MS/MS spectra of glycopeptides from NUP153 (Ser624 and Thr632 as the O-GlcNAc sites) (A), EMSY (Thr273 and Thr274 as the O-GlcNAc sites) (B), LMNA (Ser603 and Ser612 as the O-GlcNAc sites) (C) were provided.

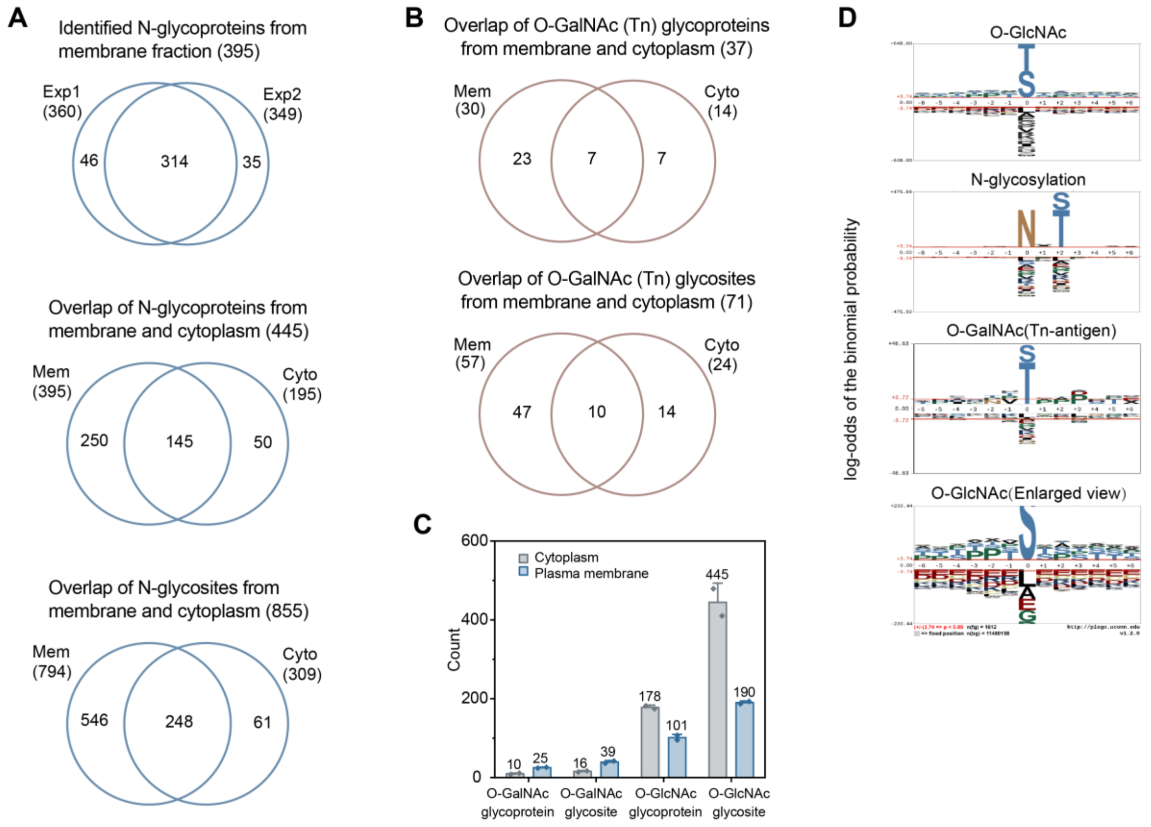

Fig. S11. Comprehensive profiling of N-glycosites, O-GlcNAc sites, and O-GalNAc sites. (A) Venn diagrams of N-glycoproteins identified by 2 replicates from membrane fraction, and N-glycoproteins/N-glycosites identified from membrane and cytoplasm. (B) Venn diagrams of total Tn-antigen glycoproteins and Tn-antigen glycosites identified from membrane and cytoplasm. (C) Numbers of O-GlcNAc/O-GalNAc sites and O-GlcNAc/O-GalNAc proteins identified from membrane and cytoplasm samples. Error bars represent mean ± *s.d.* from two independent experiments. Average values are shown above the bars. (D) Sequence analysis around the N-glycosites, O-GlcNAc sites, and O-GalNAc sites.

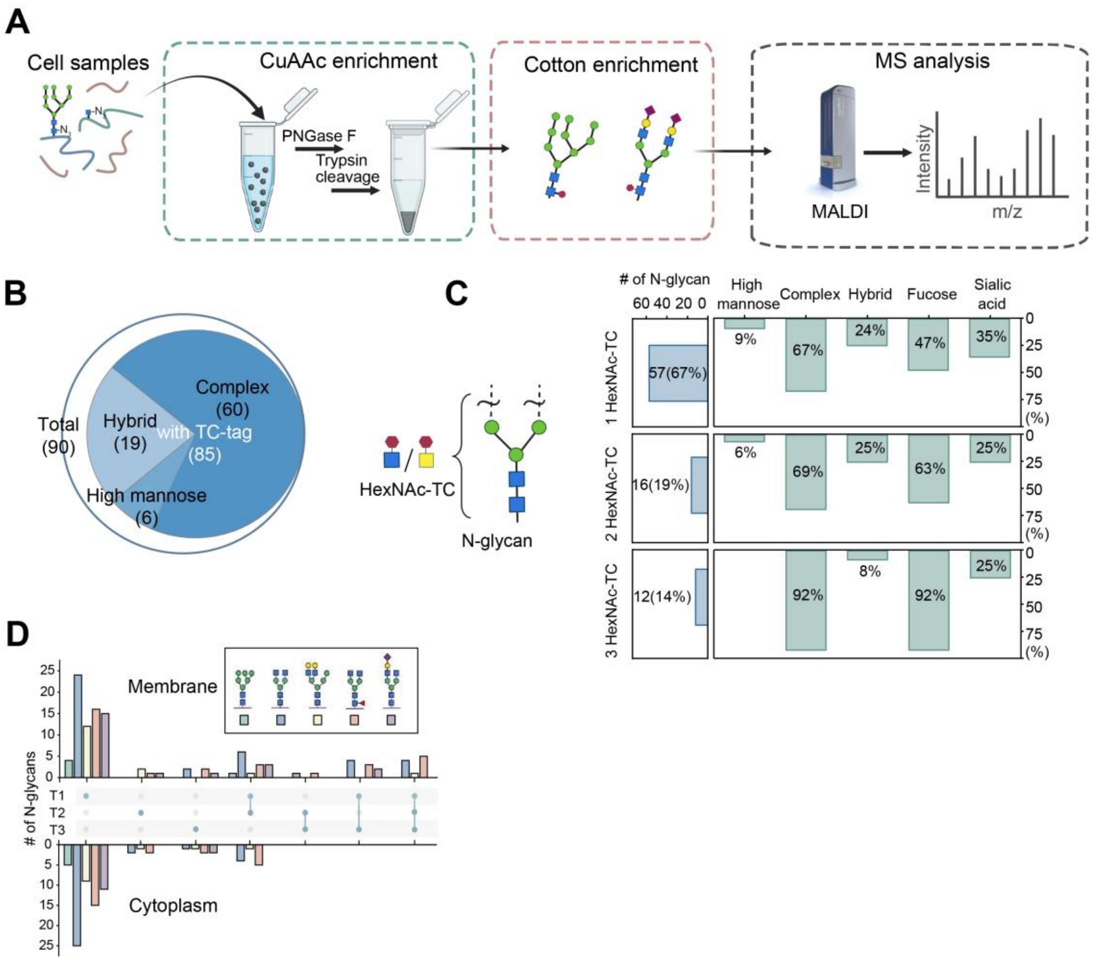

Fig. S12. Comprehensive profiling of the N-glycome identified with TC-tag. (A) Workflow of the N-glycan enrichment. (B) Distribution of N-glycans with specific types identified from membrane samples. (C) The proportion of N-glycans with specific types and TC-tag number. HexNAc-TC, glycan with TC-tag. (D) Comparison of N-glycans with different specific types and TC-tag number identified from membrane and cytoplasm.

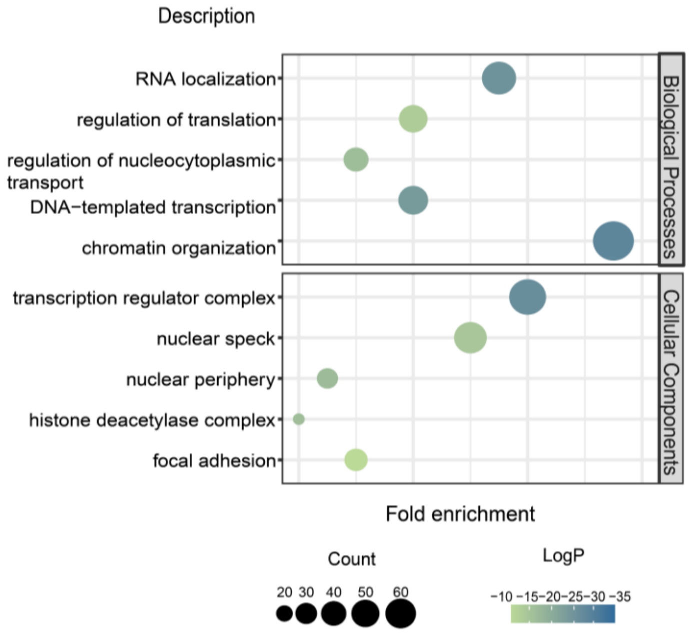

Fig. S13. GO analysis of identified O-GlcNAc glycoproteins based on biological process and cellular components.

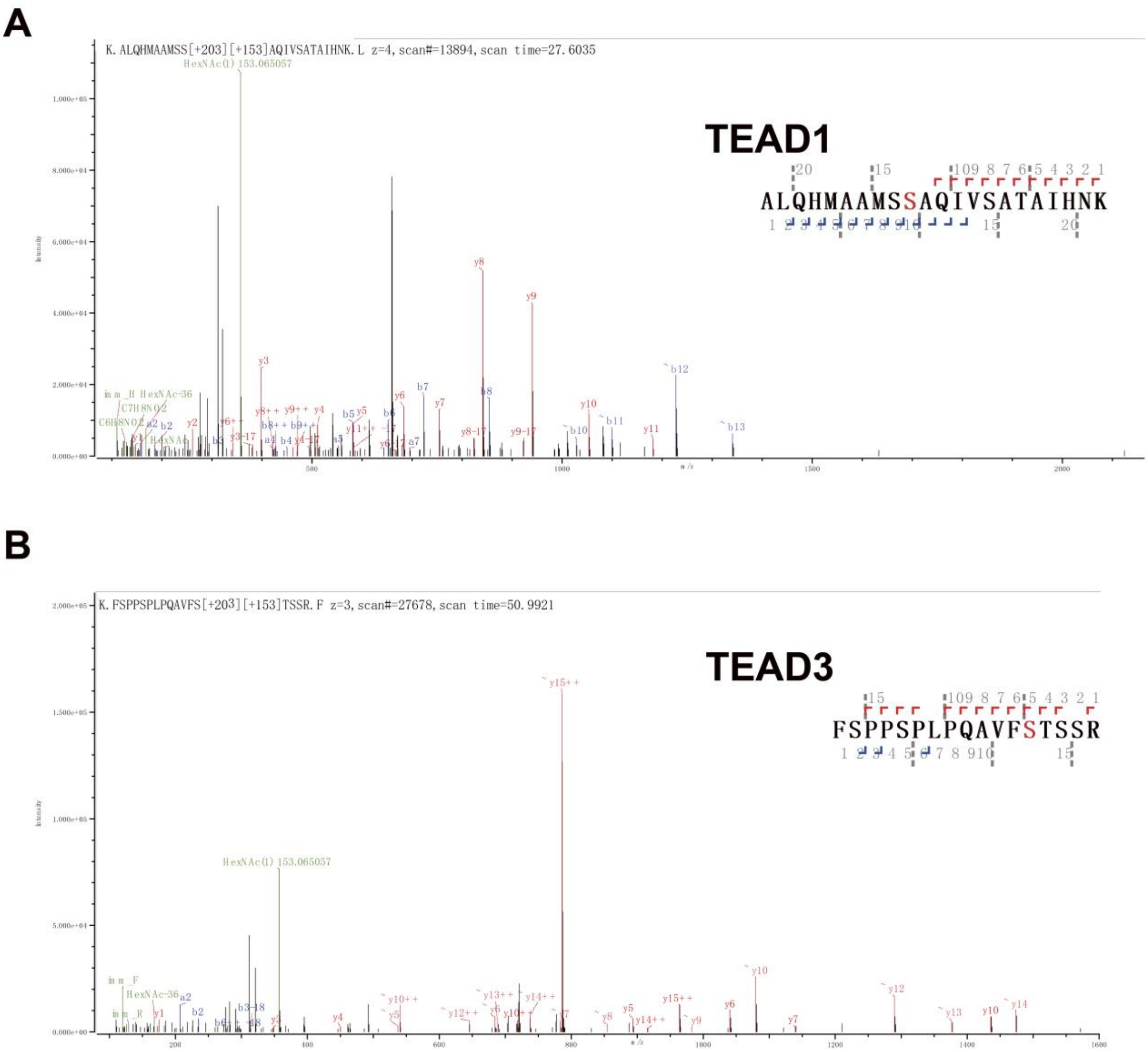

Fig. S14. HCD MS/MS spectra of glycopeptide from TEAD1 (Ser127 as the O-GlcNAc site) (A) and TEAD3 (Ser156 as the O-GlcNAc site) (B).

A

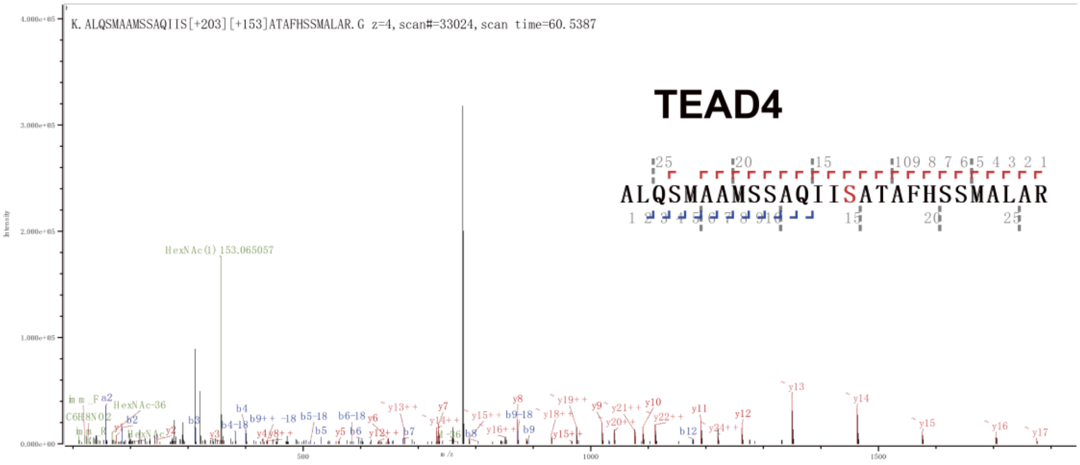

B

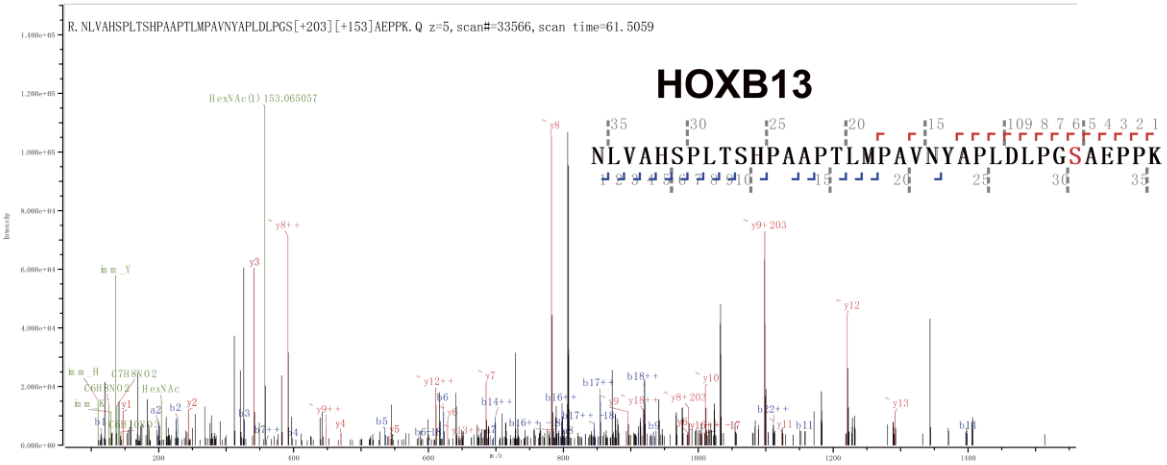

Fig. S15. HCD MS/MS spectra of glycopeptide from TEAD4 (Ser140 as the O-GlcNAc site) (A) and HOXB13 (Ser56 as the O-GlcNAc site) (B).

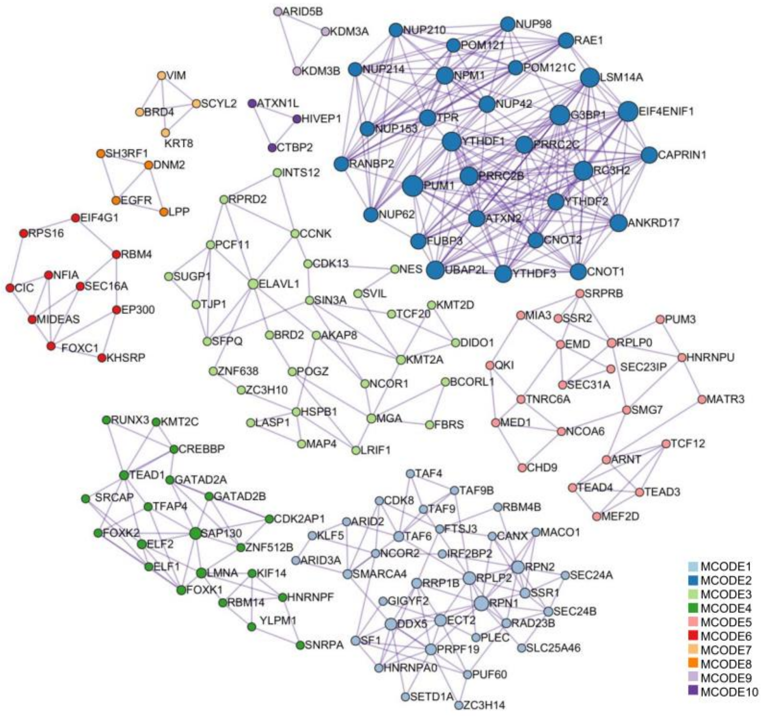

Fig. S16. Interaction network of selected 158 identified O-GlcNAc proteins in this work. The interactions were annotated in Table S7. Each node stands for a protein, and each edge represents a known protein-protein interaction.

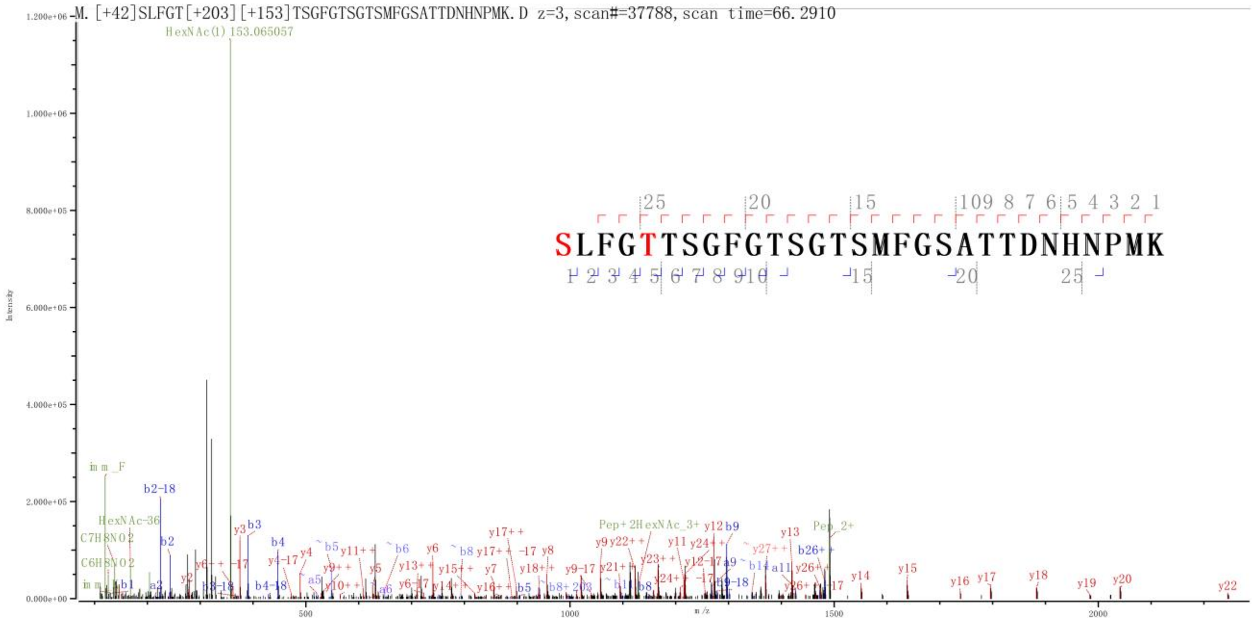

Fig. S17. HCD MS/MS spectrum of glycopeptide from RAE1 (Thr5 as the O-GlcNAc site).

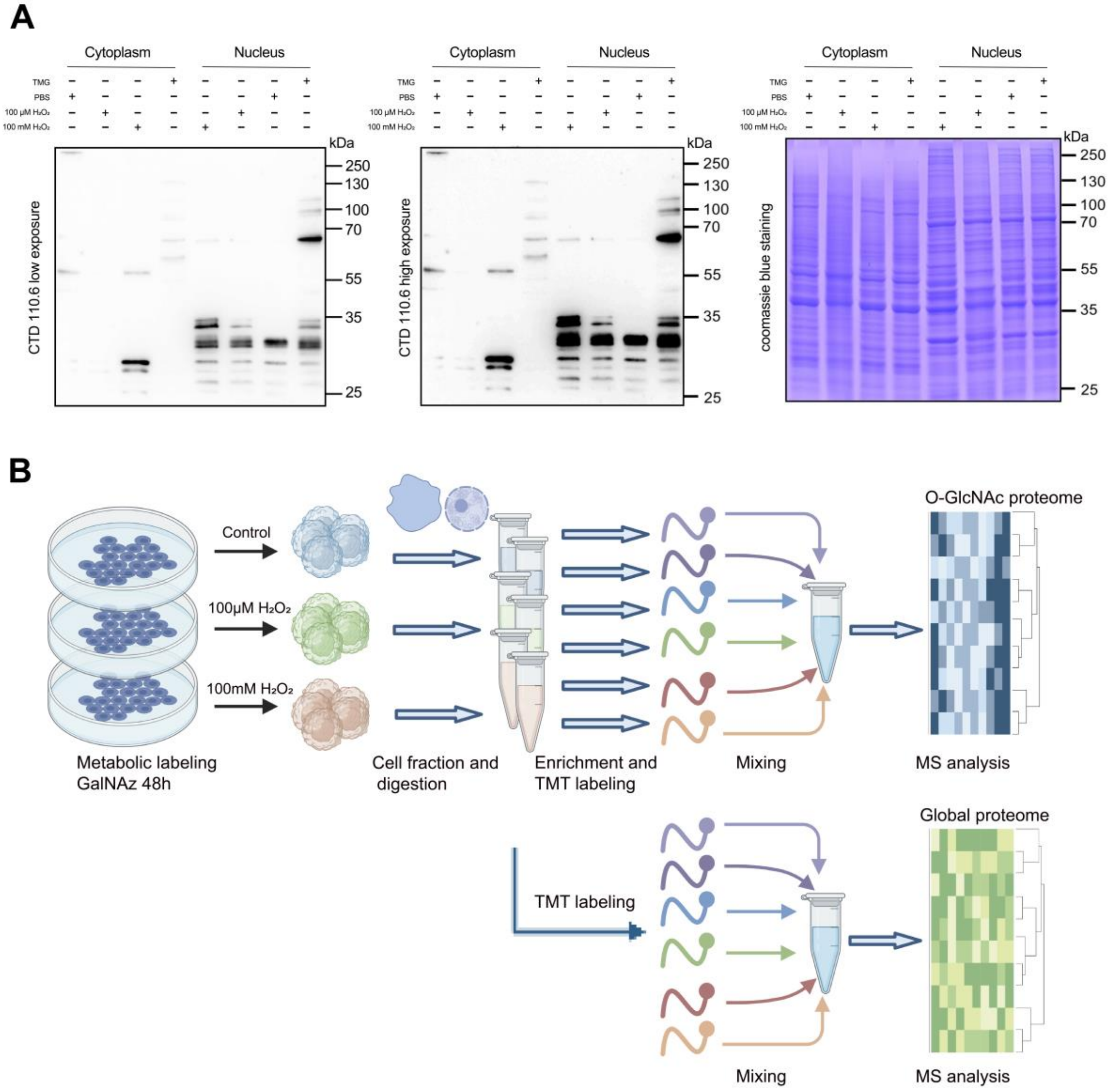

Fig. S18. Western blotting and quantitative proteomics analysis of the spatial O-GlcNAcylation patterns during oxidative stress. (A) The western blotting (detected by clone CTD110.6) and coomassie blue staining for samples from different compartments with 100 μM H_2O_2 and 100 mM H_2O_2 treatment. CTD 110.6, O-GlcNAc antibody. TMG: Thiamet G, an O-GlcNAcase inhibitor. In this experiment, we used TMG-treated samples as the positive control for reflecting changes in O-GlcNAcylation levels. (B) Experimental workflow for quantitative analysis of proteins and the O-GlcNAc glycoproteins in the nucleus and cytoplasm upon oxidative stress.

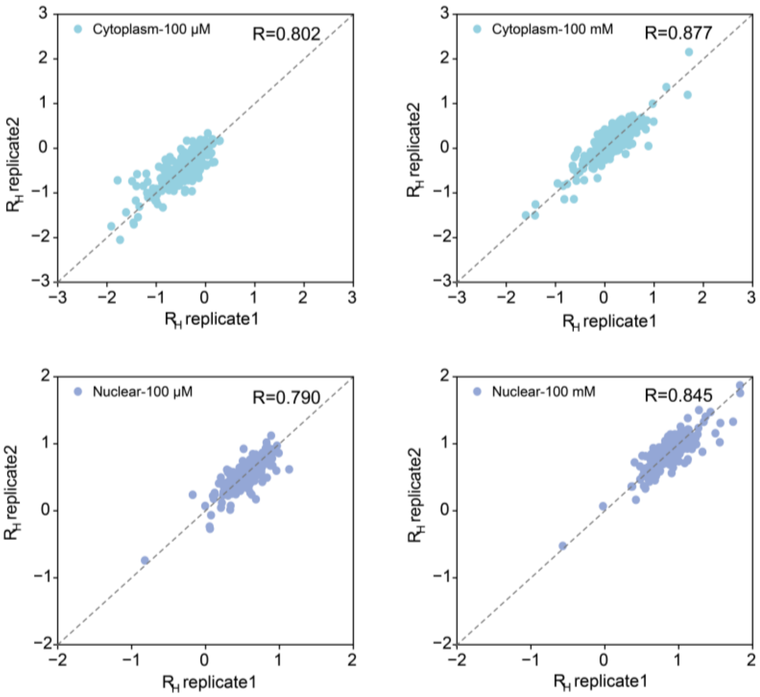

Fig. S19. Correlations of R_H value of the quantified O-GlcNAcylated peptides in the duplicate experiments. R_H: $\log_2$ (H_2O_2 treatment/control) ratio. R: Pearson *R*.

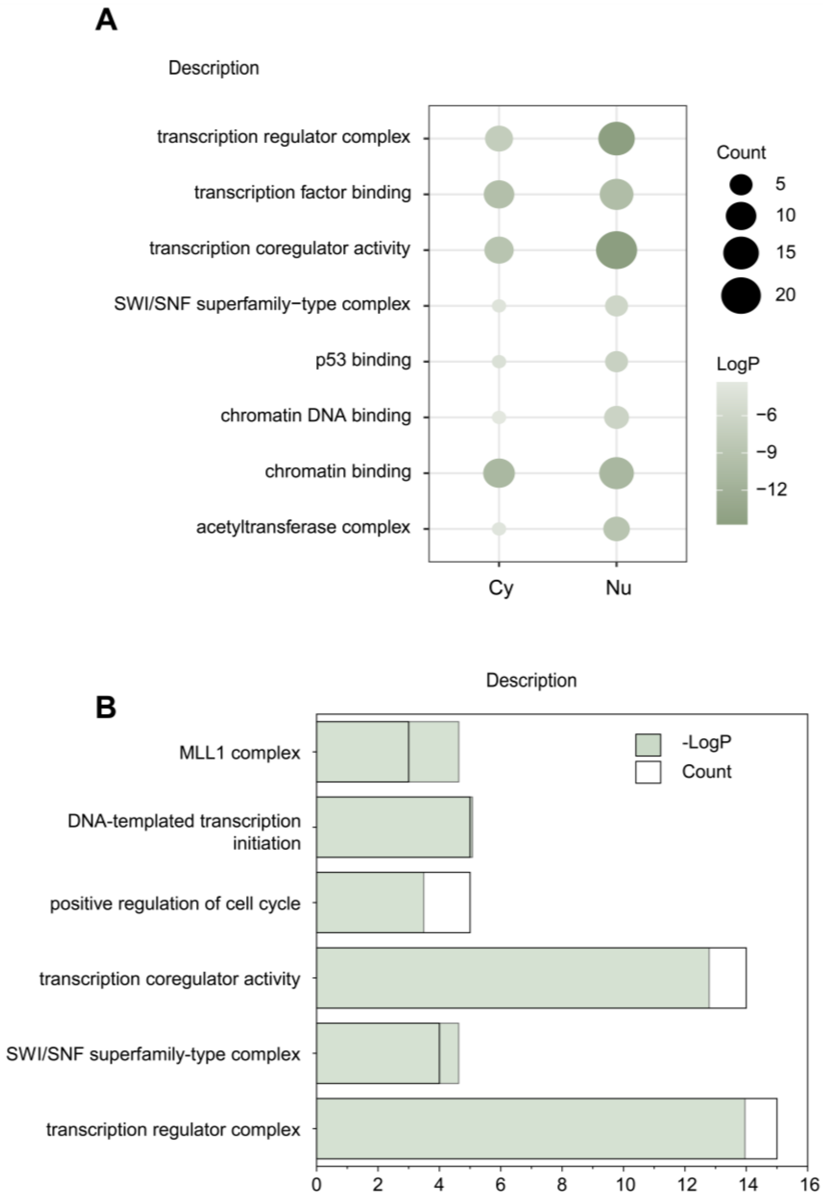

Fig. S20. Spatial-resolved investigation of O-GlcNAc protein levels in response to oxidative stress. (A) Comparison of the GO terms for glycoproteins that have significantly changed in the nucleus and cytoplasm under 100 μM H_2O_2 based on cellular compartment and molecular function. (B) GO analysis of the O-GlcNAc proteins displayed both nuclear and cytoplasmic significantly up-regulation.

Supplementary excel table titles and legends

All supplementary tables are included in the SUPPLEMENTARY DATA.

Table S1. List of O-GlcNAc glycopeptides identified from HeLa nuclear samples under different amounts of resin (from 10, 20 to 30 µL) and different peptide concentrations (from 0.5, 1.0 to 5.0 µg/µL) (enclosed excel file)

Table S2. List of O-GlcNAc glycopeptides identified from HeLa nuclear samples by biological triplicate experiments (enclosed excel file)

Table S3. The total O-GlcNAc glycosites and glycoproteins identified in HeLa cells by triplicate experiments and the overlap with two O-GlcNAc databases (enclosed excel file)

Table S4. List of N-glycopeptides identified from membrane and cytoplasm samples by replicate experiments (enclosed excel file)

Table S4. List of N-glycopeptides identified from membrane and cytoplasm samples by replicate experiments (enclosed excel file)

Table S5. List of O-GalNAc glycopeptides identified from membrane and cytoplasm samples by replicate experiments (enclosed excel file)

Table S6. List of O-GlcNAc glycopeptides identified from membrane and cytoplasm samples by replicate experiments (enclosed excel file)

Table S7. The total N-glycosites and glycoproteins identified from membrane and cytoplasm samples (enclosed excel file)

Table S8. The total O-GalNAc glycosites and glycoproteins identified from membrane and cytoplasm samples s (enclosed excel file)

Table S9. The total O-GlcNAc glycosites and glycoproteins identified from membrane and cytoplasm samples (enclosed excel file)

Table S10. List of N-glycans identified from membrane and cytoplasm samples (enclosed excel file)

Table S11. List of transcription factors identified in this work and the protein-protein interaction annotation (enclosed excel file)

Table S12. List of quantified glycopeptides after treated with H_2O_2 combining GlcNAc-ID and TMT labelling (enclosed excel file)

Table S13. List of quantified glycoproteins after treated with H_2O_2 combining GlcNAc-ID and TMT labelling (enclosed excel file)

Table S14. List of quantified glycoproteins and glycosites for analysis (enclosed excel file)

References

1. Martinez-Val A, Bekker-Jensen DB, Steigerwald S *et al.* Spatial-proteomics reveals phospho-signaling dynamics at subcellular resolution. *Nature Communications*. 2021; **12**(1). doi: 10.1038/s41467-021-27398-y

2. Peng Y, Lv J, Yang L *et al.* A streamlined strategy for rapid and selective analysis of serum N-glycome. *Analytica Chimica Acta*. 2019; **1050**: 80-87. doi: 10.1016/j.aca.2018.11.002

3. Zecha J, Satpathy S, Kanashova T *et al.* TMT Labeling for the Masses: A Robust and Cost-efficient, In-solution Labeling Approach. *Molecular & cellular proteomics : MCP*. 2019; **18**(7): 1468-1478. doi: 10.1074/mcp.TIR119.001385

4. Liu J, Cheng B, Fan X *et al.* Click-iG: Simultaneous Enrichment and Profiling of Intact N-linked, O-GalNAc, and O-GlcNAcylated Glycopeptides. *Angewandte Chemie-International Edition*. 2023; **62**(36). doi: 10.1002/anie.202303410

5. Tripathi S, Pohl MO, Zhou Y *et al.* Meta- and Orthogonal Integration of Influenza "OMICs'' Data Defines a Role for UBR4 in Virus Budding. *Cell Host & Microbe*. 2015; **18**(6): 723-735. doi: 10.1016/j.chom.2015.11.002

6. O'Shea JP, Chou MF, Quader SA *et al.* pLogo: a probabilistic approach to visualizing sequence motifs. *Nature Methods*. 2013; **10**(12): 1211-+. doi: 10.1038/nmeth.2646

7. Lambert SA, Jolma A, Campitelli LF *et al.* The Human Transcription Factors (vol 172, pg 650, 2018). *Cell*. 2018; **175**(2): 598-599. doi: 10.1016/j.cell.2018.09.045

8. Wulff-Fuentes E, Berendt RR, Massman L *et al.* The human O-GlcNAcome database and meta-analysis. *Scientific Data*. 2021; **8**(1). doi: 10.1038/s41597-021-00810-4

9. Ma J, Li Y, Hou C, Wu C. O-GlcNAcAtlas: A database of experimentally identified O-GlcNAc sites and proteins. *Glycobiology*. 2021; **31**(7): 719-723. doi: 10.1093/glycob/cwab003
